# Supplementary figures and images for: Integrins regulate epithelial cell shape by controlling the architecture and mechanical properties of basal actomyosin networks
Source: PLoS Genet. 2020 Jun 1;16(6):e1008717. doi: 10.1371/journal.pgen.1008717 (PMC7263567; doi:10.1371/journal.pgen.1008717)

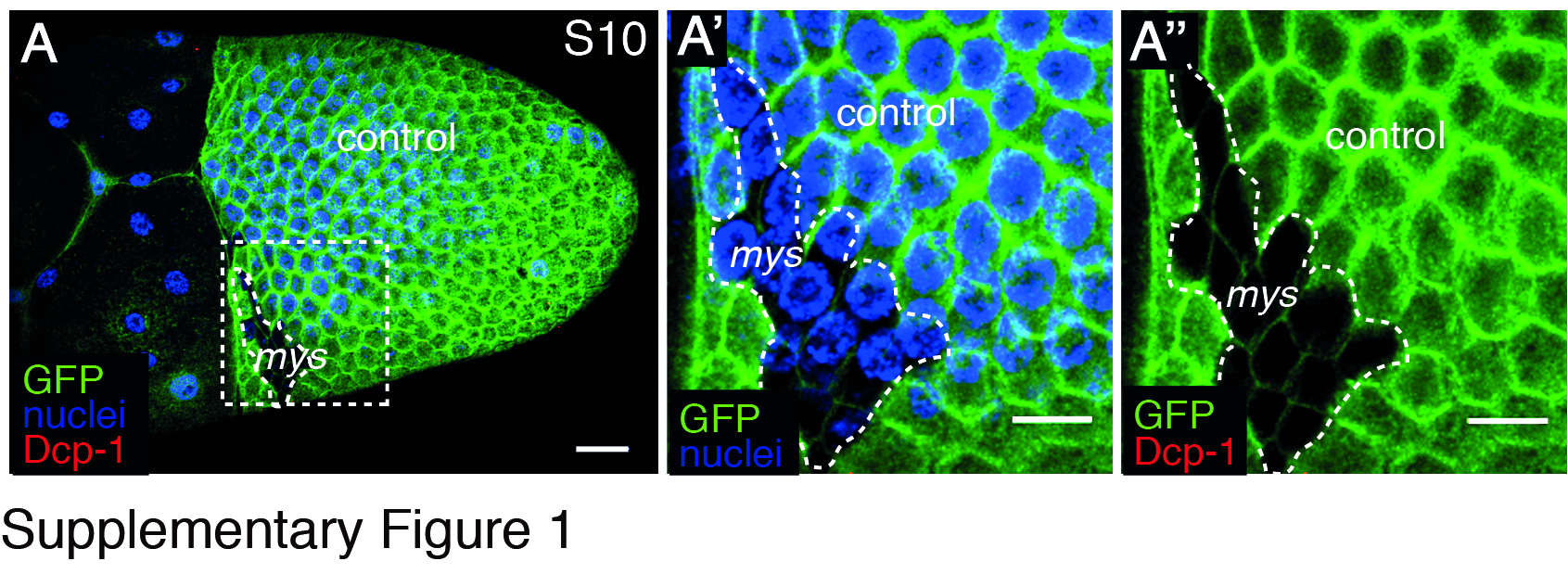

Supplement: S1 Fig — (A) Basal surface view of a mosaic S10 egg chamber containing mys FC clones stained with anti-GFP (green), anti-Dcp-1 (red) and the nuclear marker Hoechst (blue). Scale bar, 20 μm. (A’, A”) Magnifications of the white box in A. Scale bars, 10 μm. (TIF) [file pgen.1008717.s011.tif]

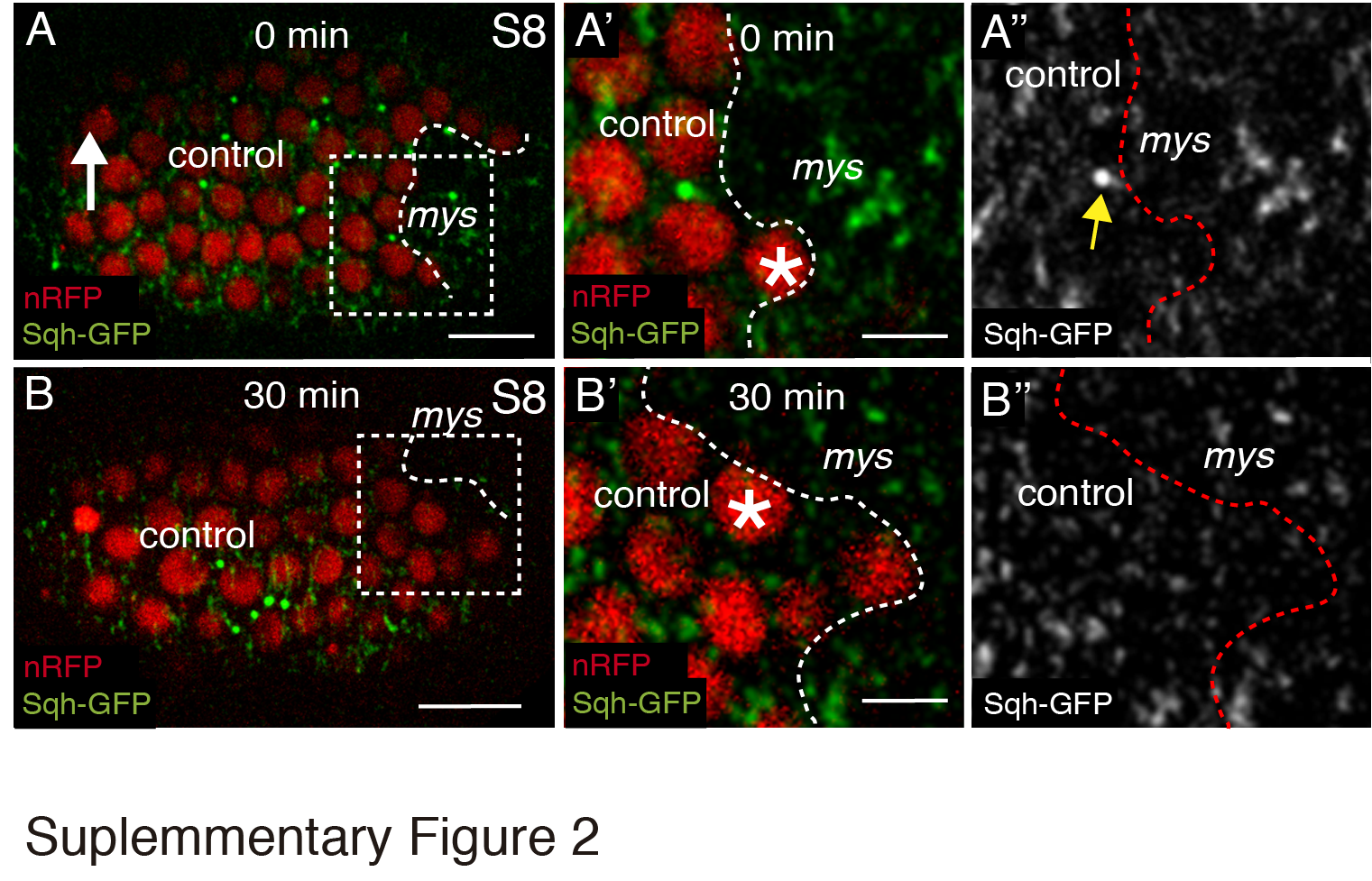

Supplement: S2 Fig — (A, B) Confocal images, taken with a 30 min. interval, of a live rotating S8 mosaic egg chamber containing mys FC clones (nuclear RFP-negative) and expressing Sqh-GFP (green). Arrow in A indicates the direction of egg chamber rotation. (A’, A”, B’ and B”) Magnifications of the white boxes in A and B, respectively. Asterisks label a cell as a reference for the rotation. Dots correspond to aggregates of the Sqh-GFP protein (yellow arrow in A”). Scale bars, 20μm in A and B and 5μm in A’, A”, B’ and B”. (TIF) [file pgen.1008717.s012.tif]

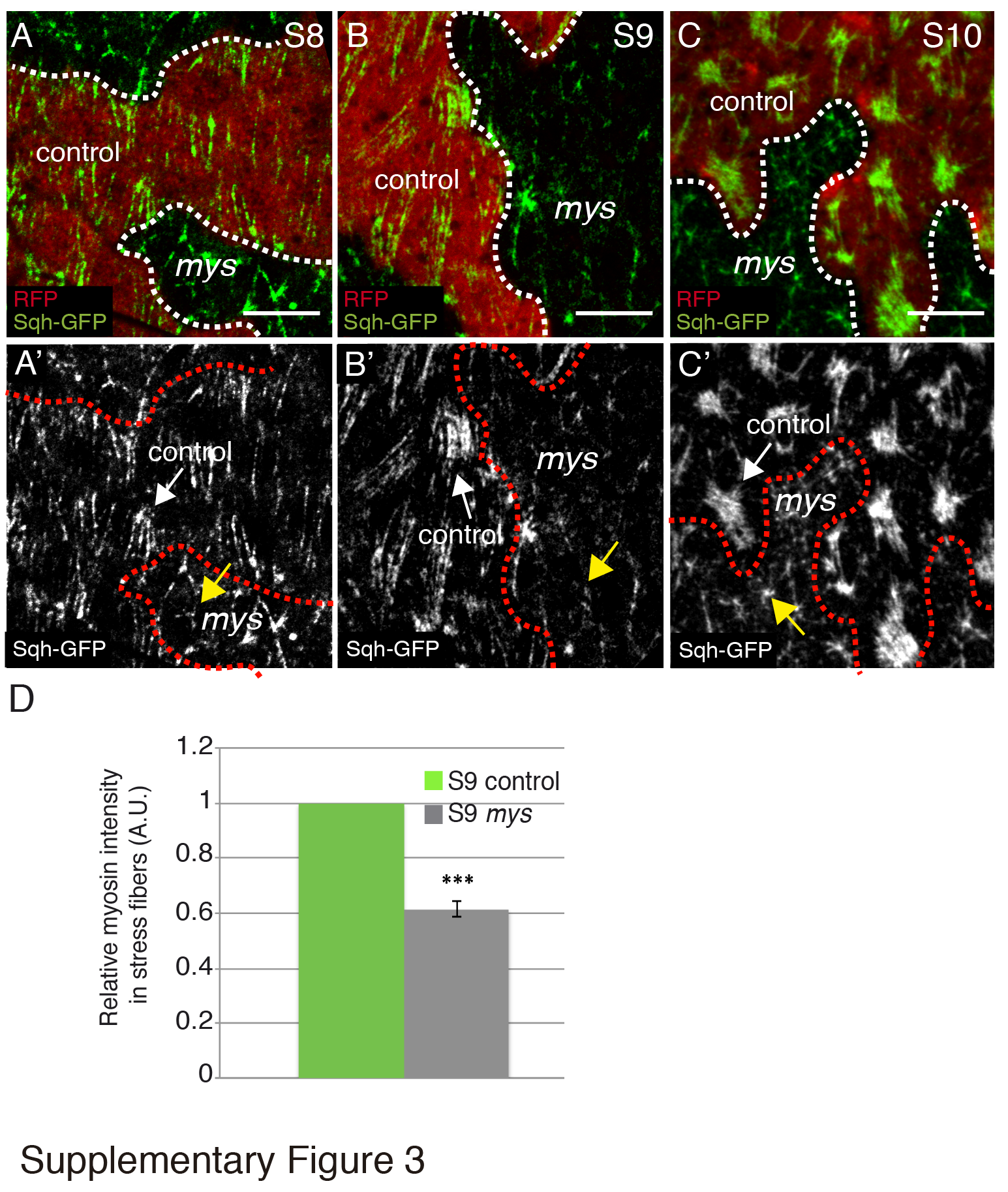

Supplement: S3 Fig — (A, B, C) Basal surface view of mosaic S8 (A, A’), S9 (B, B’) and S10 (C, C’) egg chambers containing mys FC clones, expressing Sqh-GFP (green) and stained for anti-RFP (red). (A-C’) Myosin levels in stress fibers diminish progressively from S8-10 in mys FCs (RFP-negative). White and yellow arrows point to stress fibers in control (RFP-positive) and mutant FCs, respectively. (D) Quantification of relative myosin levels in stress fibers in control and mys FCs. Scale bars, 5μm. (TIF) [file pgen.1008717.s013.tif]

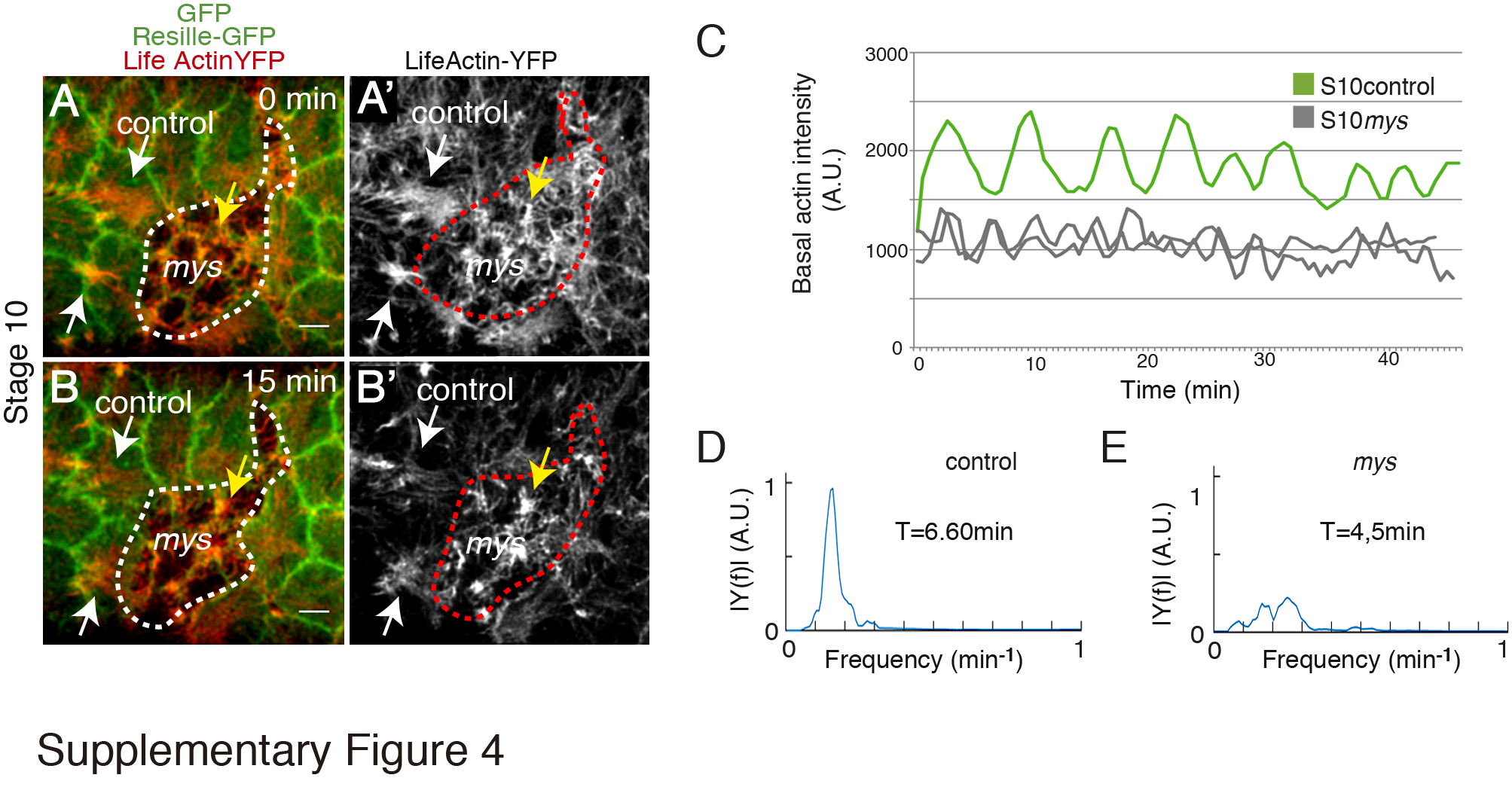

Supplement: S4 Fig — (A, B’) Confocal images, taking with a 15 min interval, of live S10 egg chambers containing mys FC clones and expressing LifeactinYFP (red) and the cell membrane marker Resille-GFP (green). White and yellow arrows point to stress fibers in control (GFP-positive) and mutant (GFP-negative) FCs, respectively. (C) Quantification of the dynamic changes of basal F-actin intensity in S10 control (green) and mys FCs (grey). (D, E) Fourier transform of the autocorrelation function of the temporal sequences of basal F-actin intensity for control (D) and mys (E) FCs. T indicates period of oscillations. Scale bars, 5 μm. (TIF) [file pgen.1008717.s014.tif]

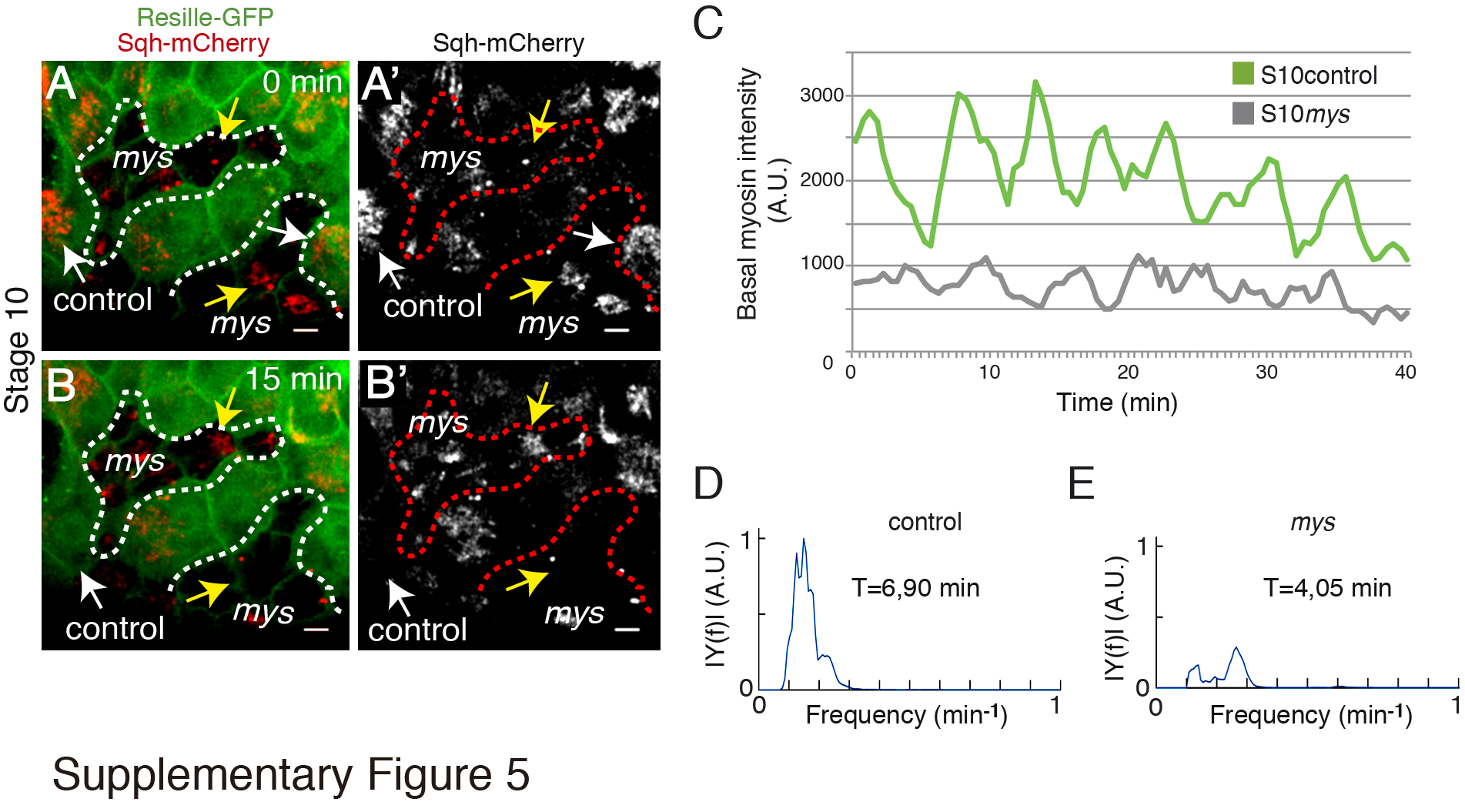

Supplement: S5 Fig — (A, B’) Confocal images, taking with a 15 min. interval, of live S10 egg chambers containing mys FC clones and expressing Sqh-mCherry (red) and the cell membrane marker Resille-GFP (green). (A’, B’) White and yellow arrows point to stress fibers within control (GFP-positive) and mutant (GFP-negative) FCs, respectively. (C) Quantification of the dynamic changes of basal myosin intensity in S10 control (green) and mys (grey) FCs. (D, E) Fourier transform of the autocorrelation function of temporal sequences of basal myosin intensity for control (D) and mys FCs (E). T indicates period of oscillations. Scale bars, 5 μm. (TIF) [file pgen.1008717.s015.tif]

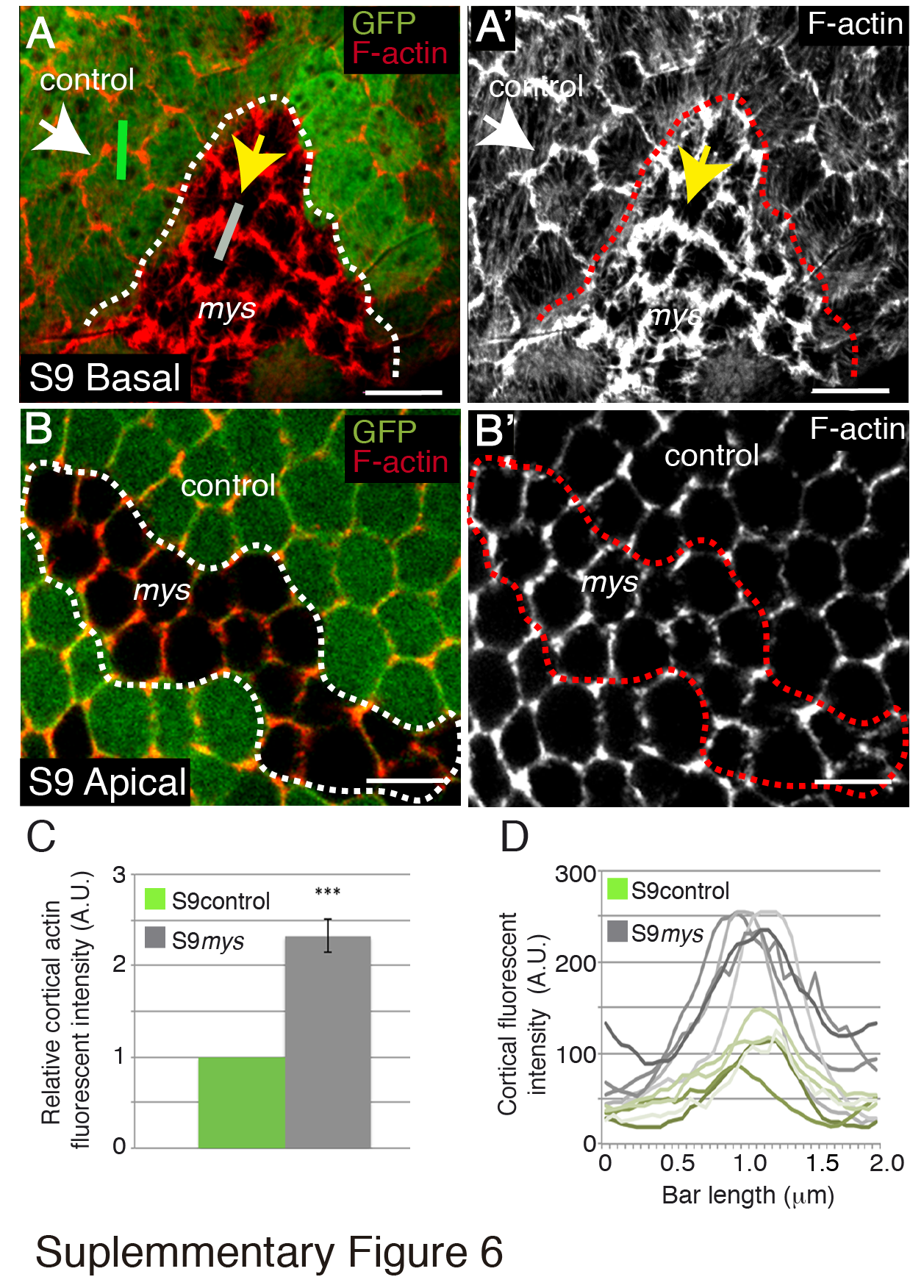

Supplement: S6 Fig — (A-B’) Basal (A, A’) and apical (B, B’) surface views of S9 mosaic egg chambers containing mys FC clones, stained for anti-GFP (green) and Rhodamine Phalloidin to detect F-actin (red). Basal (A, A’), but not apical (B, B’), cortical actin levels are higher in mys FCs (GFP-negative, yellow arrow) compared to controls (GFP-positive, white arrow). (C) Quantification of relative cortical actin intensity in control and mys FCs. (D) Histogram of fluorescent intensities of F-actin along boundaries between control and mys FCs, as indicated with straight coloured lines in (A). Scale bars, 5 μm. (TIF) [file pgen.1008717.s016.tif]

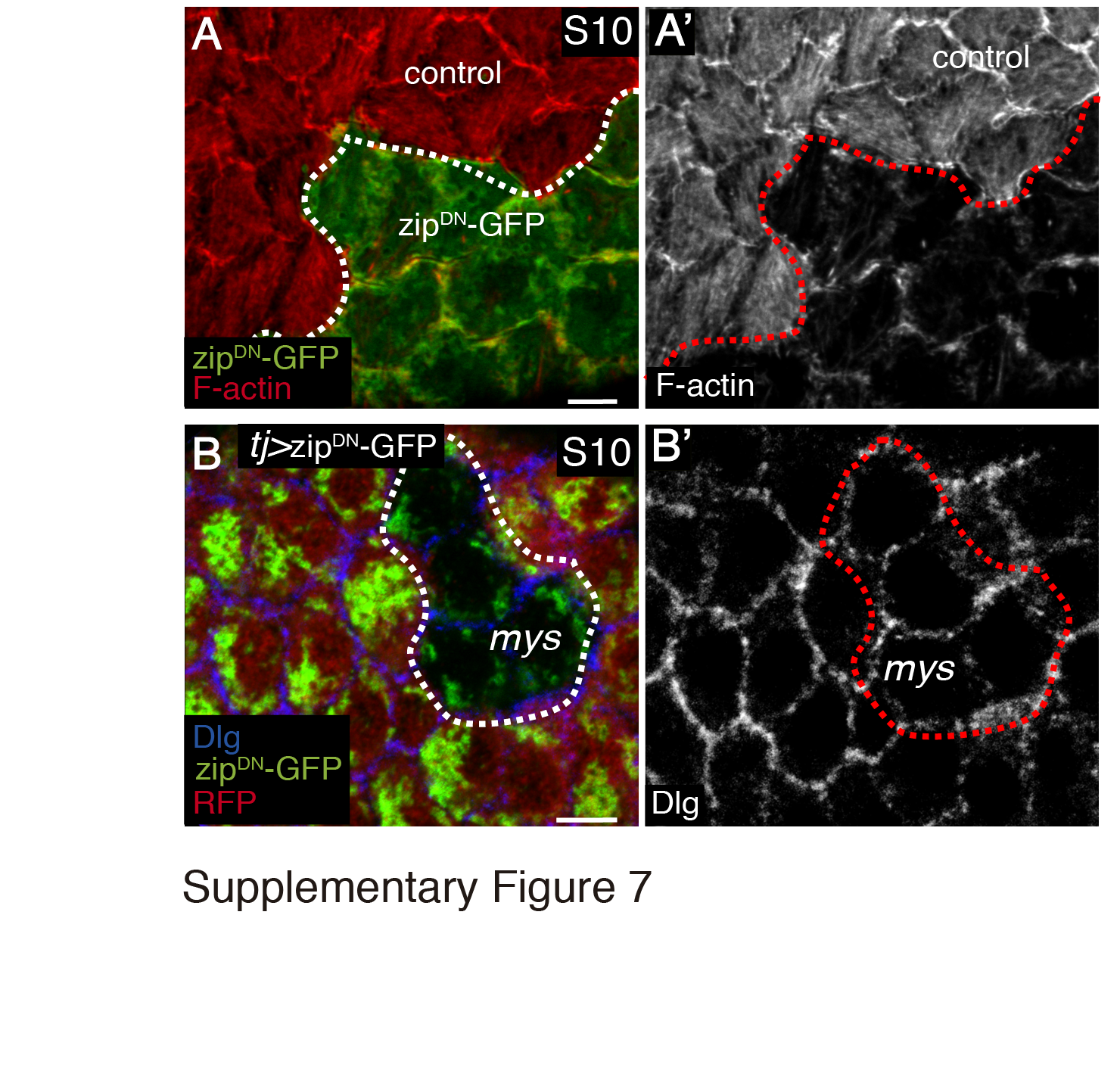

Supplement: S7 Fig — (A, A’) A S10 mosaic egg chamber containing clones of FCs expressing zipDN-GFP and stained with anti-GFP (green) and Rhodamine Phalloidin to detect F-actin (red). (B, B’) Mosaic egg chamber expressing zipDN-GFP in all FCs and containing mys FC clones (GFP-negative) stained with anti-GFP (green), Rhodamine Phalloidin (red) and anti-Dlg (blue). (TIF) [file pgen.1008717.s017.tif]

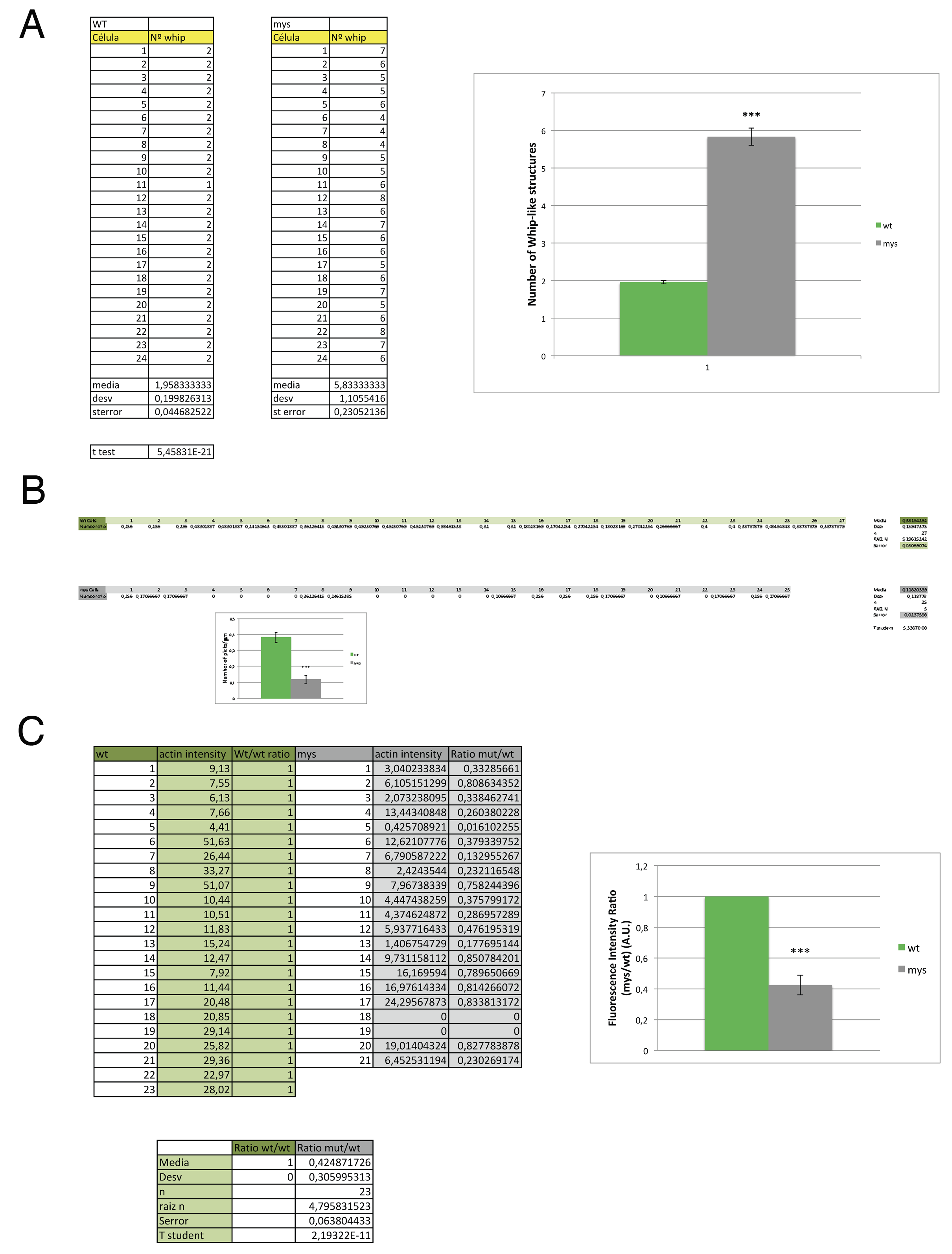

Supplement: S1 Data — (A) Whip-like structures (Fig 1F). (B) Number of peaks/μm (Fig 1G). (C) Relative actin intensity in stress fibres (Fig 1H). (TIF) [file pgen.1008717.s018.tif]

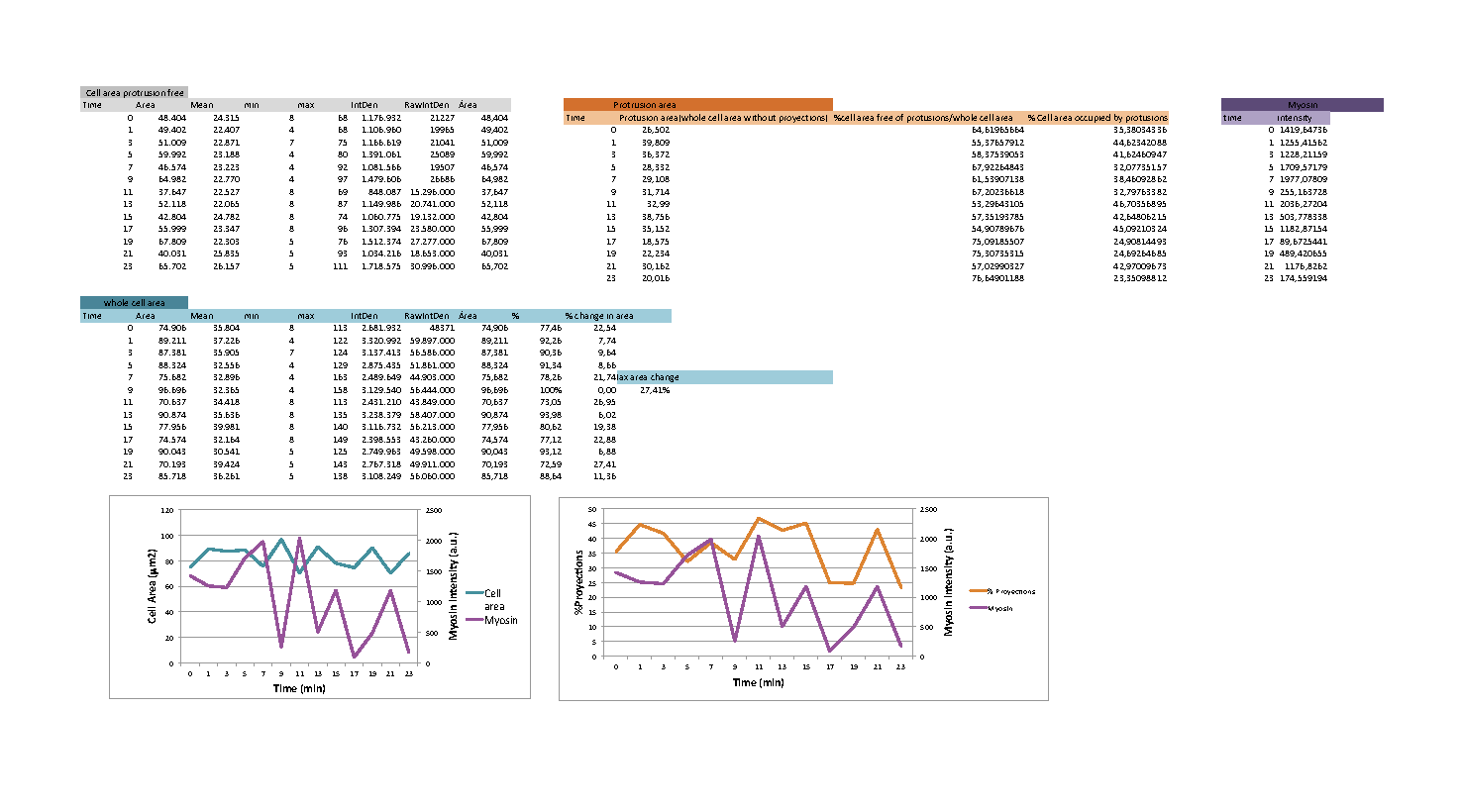

Supplement: S2 Data — Basal area occupied by projections, basal surface area, myosin intensity (Fig 2E). (TIFF) [file pgen.1008717.s019.tiff]

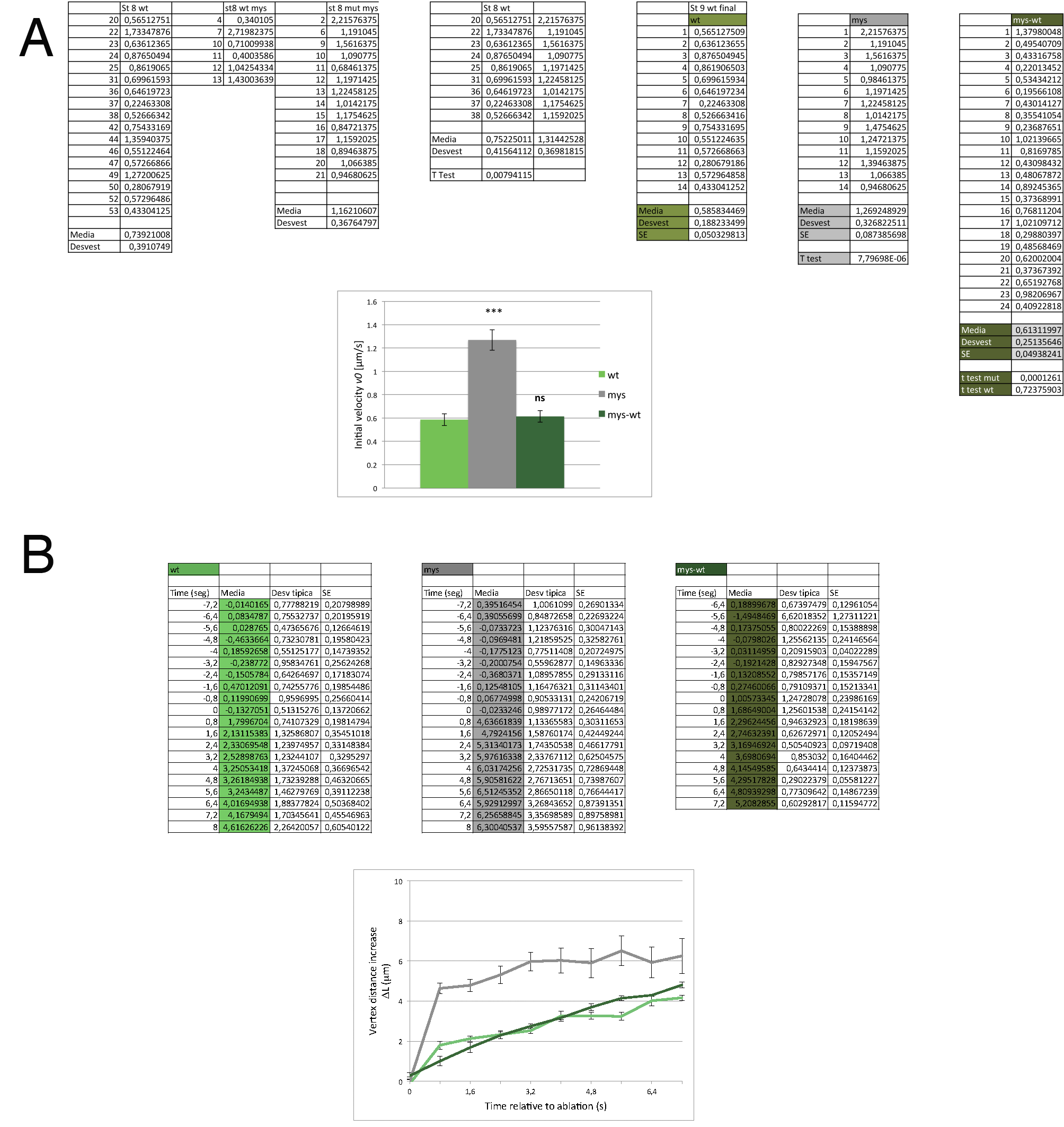

Supplement: S3 Data — (A) Initial velocity vertex displacement (Fig 3C). (B) Vertex distance increase (Fig 3D). (TIF) [file pgen.1008717.s020.tif]

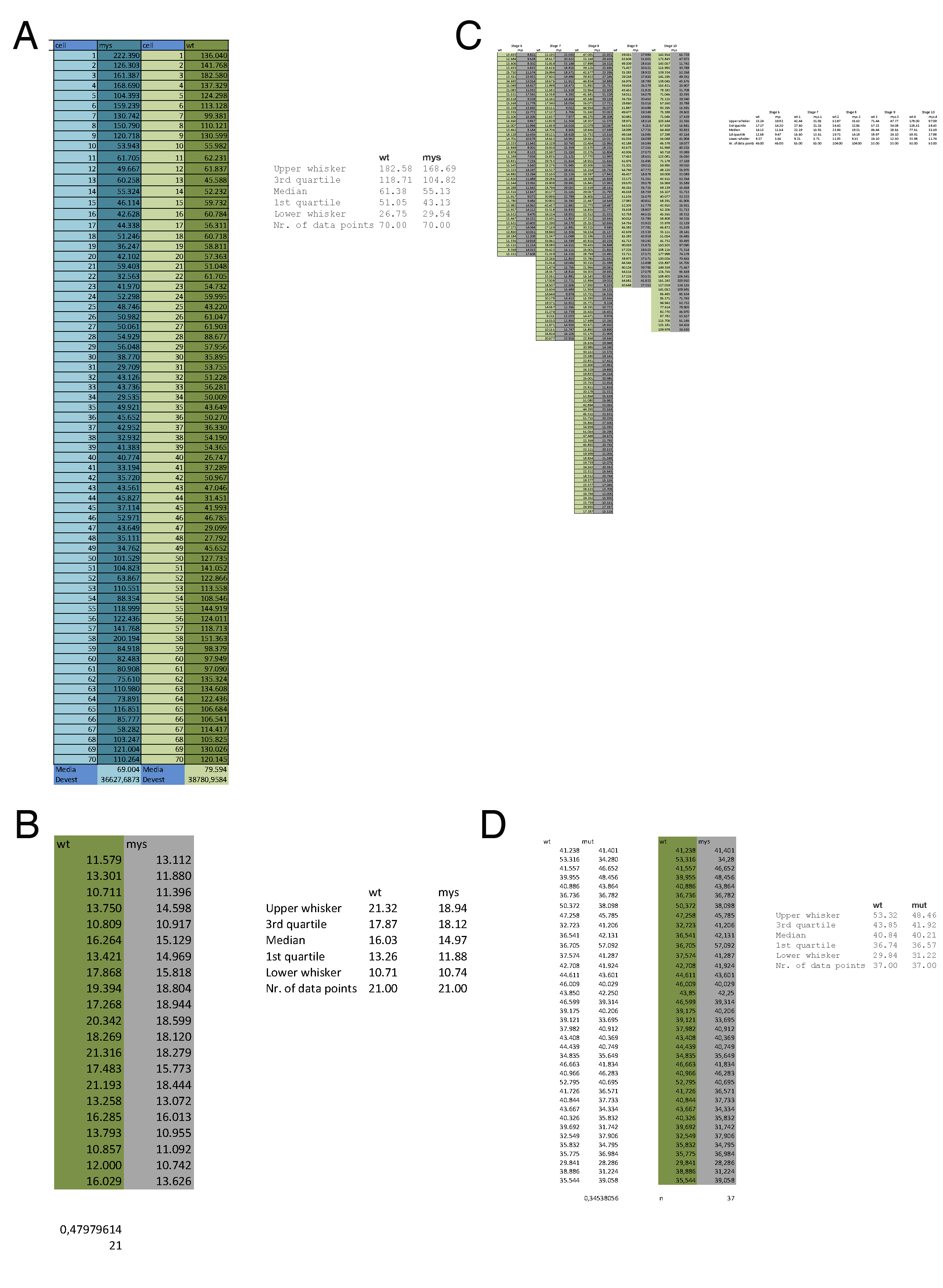

Supplement: S4 Data — (A) Apical area (Fig 4E). (B) Height (Fig 4F). (C) Basal area through development (Fig 4G). (D) Nuclear size (Fig 4H). (TIF) [file pgen.1008717.s021.tif]

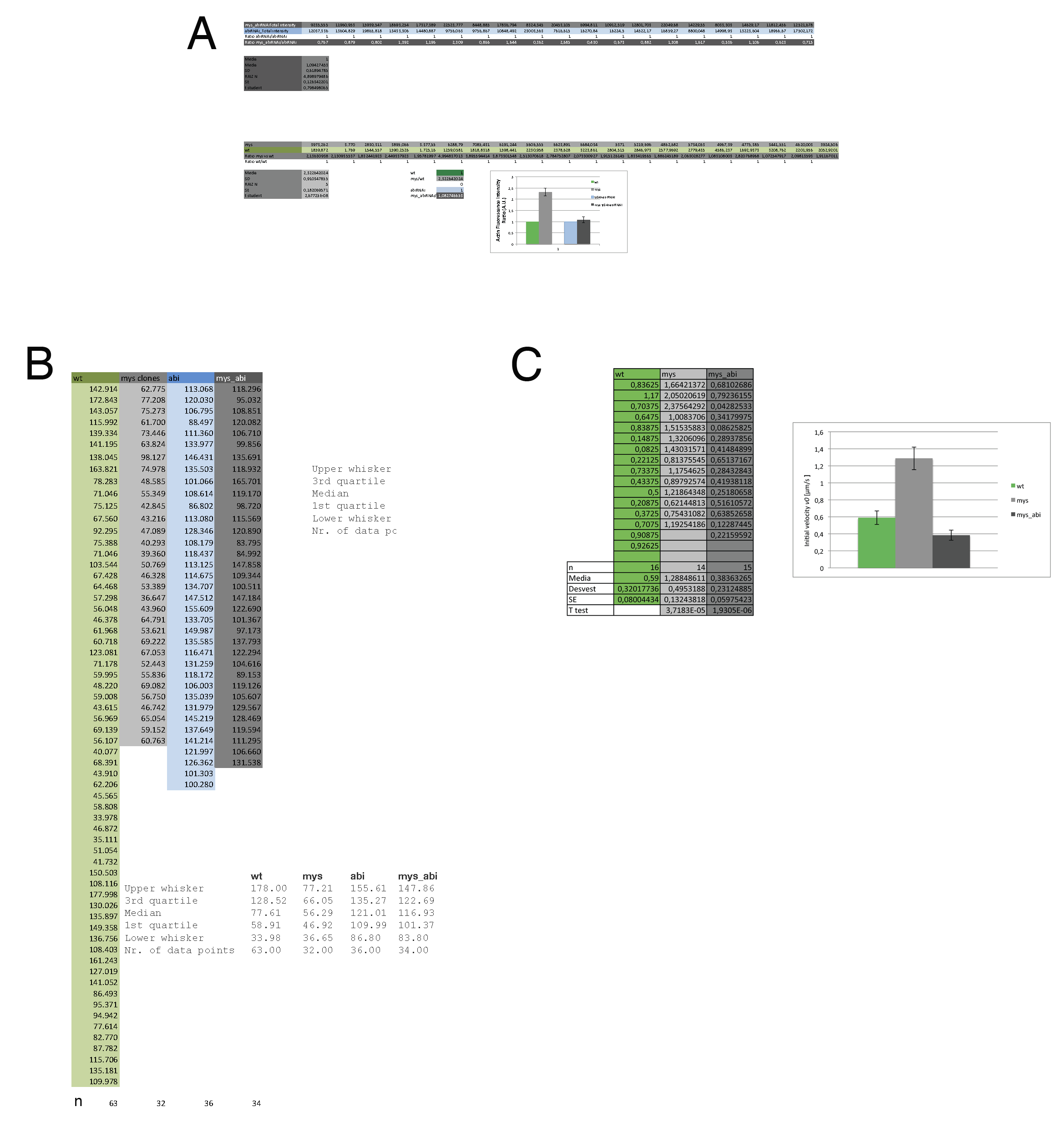

Supplement: S5 Data — (A) Cortical actin fluorescence intensity ratio (Fig 5C). (B) Basal area rescue (Fig 5D). (C) Initial Velocity vertex displacement rescue (Fig 5E). (TIF) [file pgen.1008717.s022.tif]

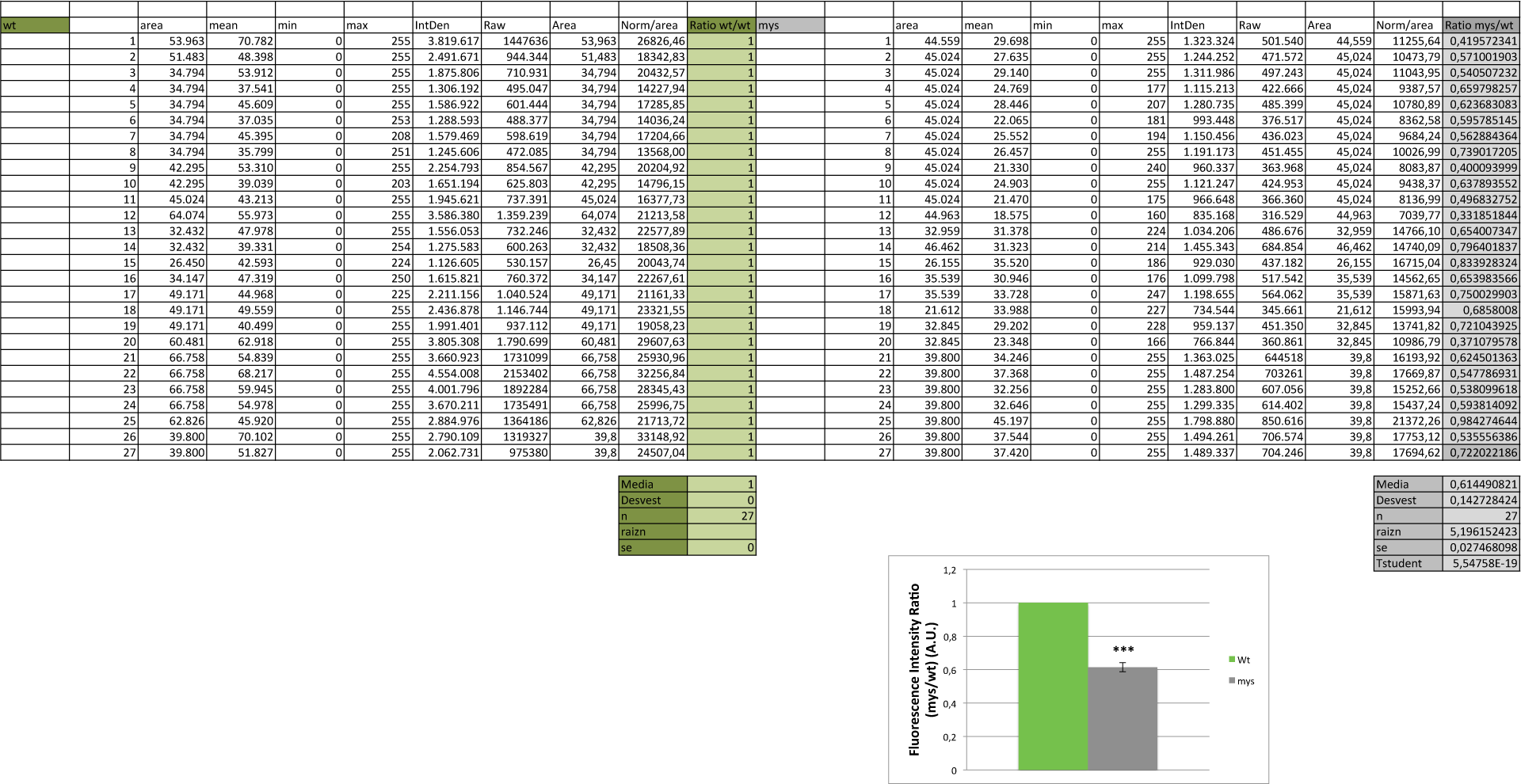

Supplement: S6 Data — Relative myosin intensity (S3D Fig). (TIF) [file pgen.1008717.s023.tif]

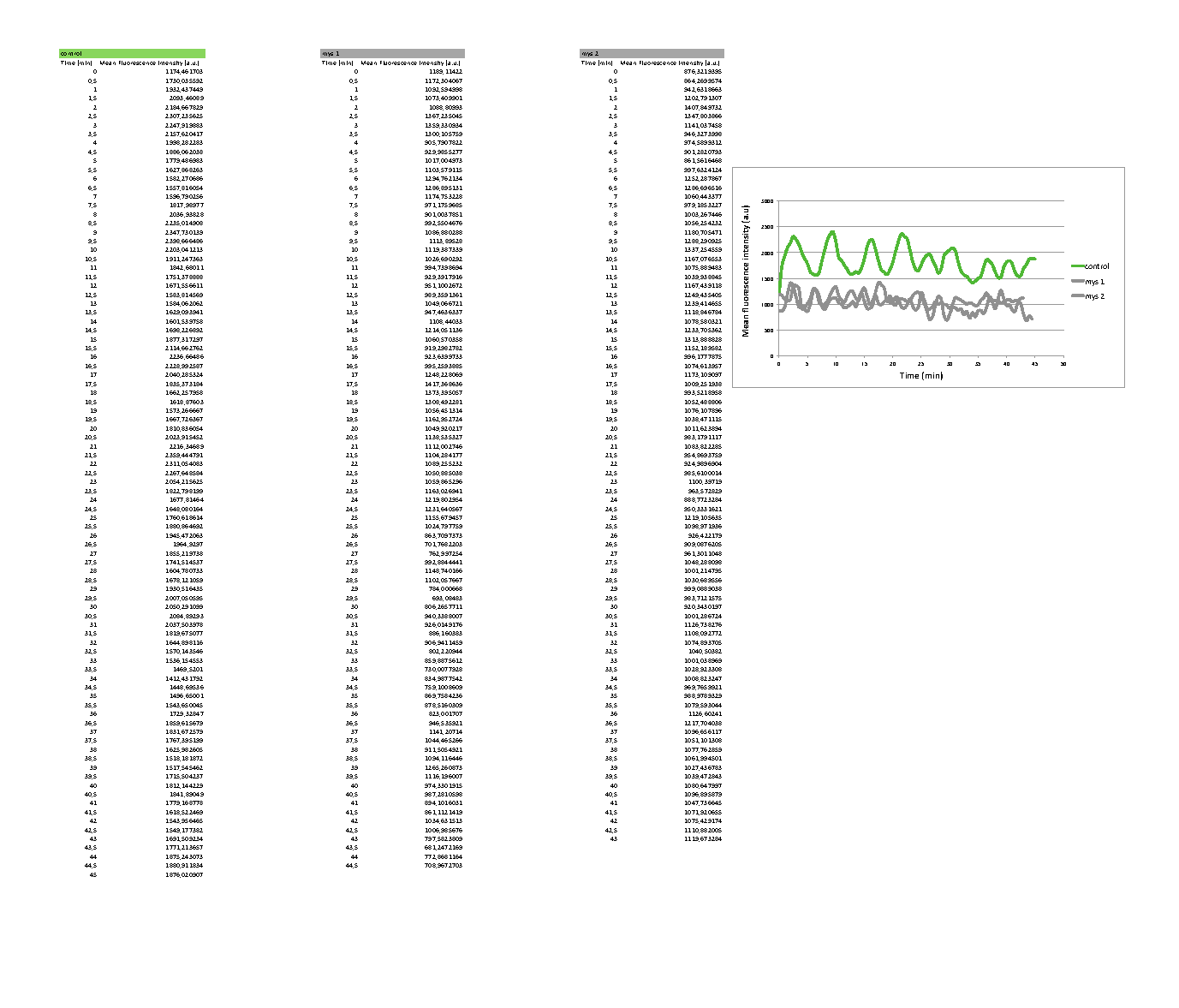

Supplement: S7 Data — Basal Actin Oscillation (S4C Fig). (TIF) [file pgen.1008717.s024.tif]

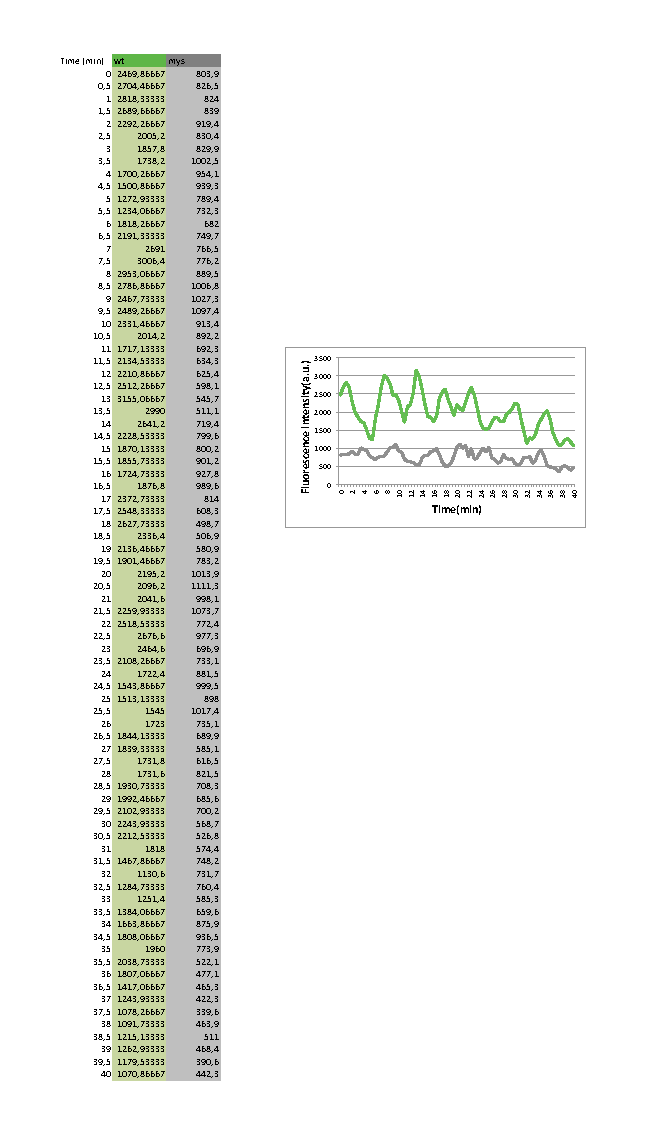

Supplement: S8 Data — Basal myosin oscillation (S5C Fig). (TIF) [file pgen.1008717.s025.tif]

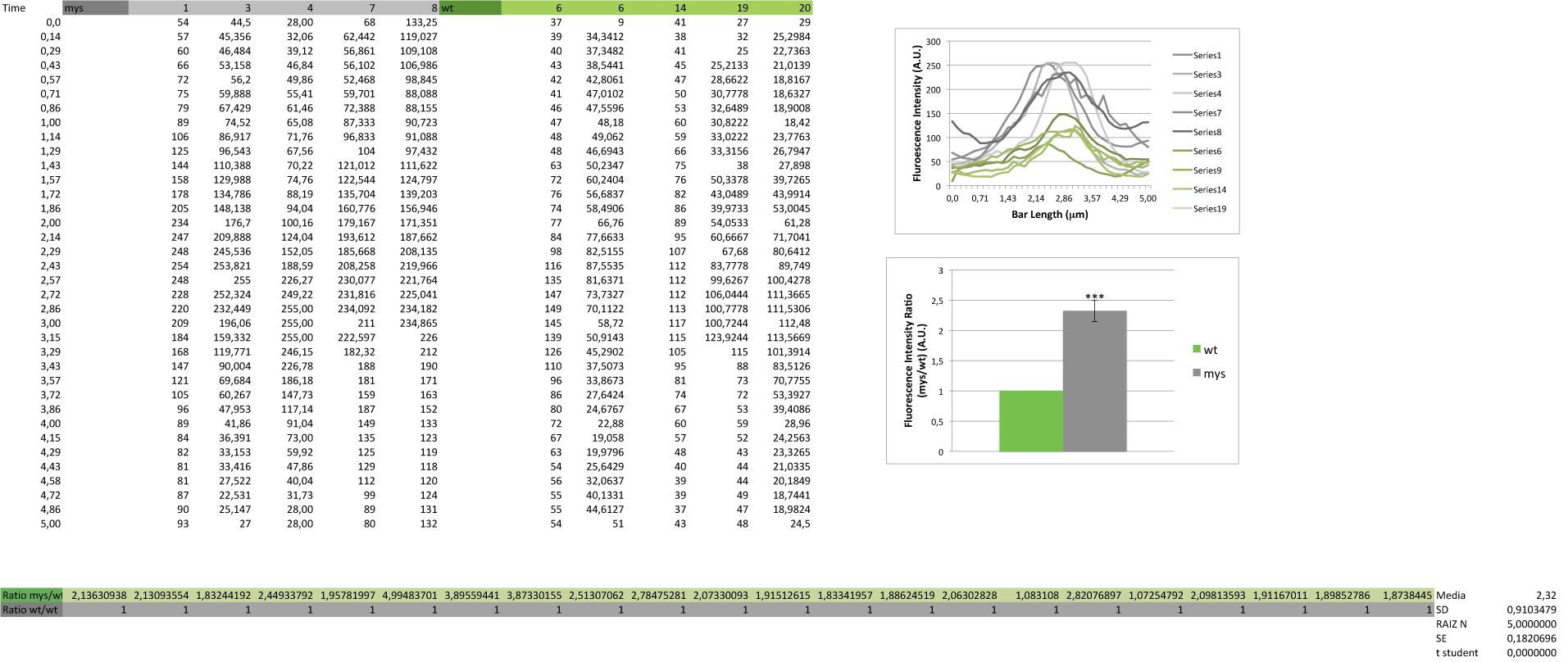

Supplement: S9 Data — Cortical actin fluorescence (S6C and S6D Fig). (TIF) [file pgen.1008717.s026.tif]
