# Supplementary material for: Integrins regulate epithelial cell shape by controlling the architecture and mechanical properties of basal actomyosin networks
Source: PLoS Genet. 2020 Jun 1;16(6):e1008717. doi: 10.1371/journal.pgen.1008717 (PMC7263567; doi:10.1371/journal.pgen.1008717)
Supplement: Attachment — Submitted filename: Manuscript Review PGENETICS.pdf [file pgen.1008717.s027.pdf]

Manuscript Review PGENETICS-D-19-01440

This manuscript investigates the role of integrins in the organization of basal actomyosin structures, specifically that of basal stress fibers, tri-junctional whips, cortical cytoskeleton. The authors conclude that loss of myospheroid (βPS subunit) successively eliminates basal stress fibres between stages 8 and 10 in Drosophila egg chambers. This loss redistributes actin into tri-junctional whips and the cortical cytoskeleton. The stronger accumulation of actin in the cortical cytoskeleton is suggested to promote enhance cortical contractility driving basal surface reduction. The shrinkage of the basal surface correlates with reorientation of neighboring wild type cells towards *mys* mutant cells and occupancy of the free basal space.

The paper proposes a really interesting idea (shift in basal actin dynamics upon mys loss of function) and follows this idea using state-of-the-art tools (imaging, quantification, genetics). The authors test this idea in a complex developmental context in vivo and have generate beautiful live imaging movies to back up their conclusions.

General comments

While I really appreciate the concept of this idea and I think that the data presented is of high quality, I have one large concern that I think needs to be addressed: I am not convinced that what the authors describe to be 'cortical actin' is indeed 'cortical actin'. The interpretation of these 'cortical' structures, however, underlies large conclusions in this manuscript and thus needs to be more clearly validated.

All images of mys mutant clones, the author describe actin structures at basal cell-cell junctions, which they claim are 'contractile cortical actin'. They base this conclusion on (1) very basal confocal sections and (2) changes in recoil velocity of basal cell-cell junctions.

I am not sure that these structures are cortical and that they can explain the change in basal contractility observed. To me these actin structures look more like basal filipodia extensions, rather than a contractile actin structure on bicellular junction. So, I have a hard time reconciling these structures with a change in basal contractility at a mechanistic level. Also, I think they do not appear to contain (more) Sqh/Myosin to explain higher levels of contractility. Also, even though the levels of these 'cortical' structures correlate with basal contractility in the functional experiments, I am not convinced that they are causative of the cortical contractile changes (rather than coregulated).

In short: I would love to see at least a better description of the 'cortical' structures (what does the basolateral cell cortex look like? Are these true intracellular actin structures or filipodia extension? Is there Myosin on those?) at the basal cell-cell junction and, ideally, better functional evidence that they cause bicellular junction contractility at the basal side.

Minor comments:

'…Here, we found that integrins also accumulated cortically, localizing with proteins associated to cortical F-actin, such as spectrins (Fig.1C, (Ng et al., 2016). Finally, integrins were also found highly enriched at tricellular junctions (arrowhead in fig.1C)….'

- Not sure what the significance of this observation is this early on in the paper. To me the localization in tricellular junctions was never really linked to any of the observations in the paper. I think it should somehow link to the tricellular whips, but that has not been spelled out.

'…However, the increase in F-actin observed in mysXG43 FCs seemed associated with the whip-like structures found at tricellular junctions rather than with the stress fibers…'

- To me the actin structures in Fig 1D look like basal actin stress fibers and not tricellular whips! Can you please show a different image that matches your description of lost stress fibres? I think this is important for Figure 1D.

'…propel contrary to the direction of egg chamber rotation (Movie 1)…'

- should this be 'propeling' ?

'…we challenged our previous model and tested if reduced F-actin levels could lead to shorten and more stochastic oscillations, as well as reduced myosin levels (Valencia-Exposito et al., 2016). We found this was the case (Fig.S3F-H), proving the robustness of the model…'

- I do not get this sentence. What model did you test and how does this prove that reduced actin levels alone (rather than lack of mys) are sufficient to shorten oscillations?

'…Adherent mouse embryonic fibroblasts that gradually detach from the substrate redistribute their F-actin from stress fibers to a more cortical position…'

- Reference for this statement is missing…

'… We found elevated levels of cortical F-actin in the mutant FCs compared to controls (Fig.3A, A')…'

- See my comment at the beginning but: How do you know that this is cortical and not just a bunch of whips? Many of the bicellular junctions do not show cortical enrichment. The section is very basal. How do you define cortical actin?

'…To do this, we generated large clones and compared cortical F-actin in mutant cells surrounded by either control or mutant cells. We found that cortical F-actin levels were increased in all mutant cells, suggesting that the defects in cortical F-actin due to elimination of integrins are cell autonomous (Fig.S5B, B')…'

- Why do you not show this large clone in the main figure? It immediately answers the question of autonomy (even though I still think that these a filipodia or whip structures and not 'cortical' F-actin).

Figure 3C

- What is the x-axis of that graph? Label is missing.

'…These results demonstrate that cell membranes are under higher tension in integrin mutant cells. Altogether, these results strongly suggest that integrins regulate basal cortical F-actin levels and cortical tension in FCs…'

- Laser ablation shows higher tension on these junctions but there is no extra myosin there. Is there more myosin on the cortical junctions to explain higher recoil? Also, the recoil is along the vector of the membrane but the filaments appear orientated perpendicular to it. How can they pull to generate higher tension?

Figure 5C

- What is the x-axis of that graph? Label is missing…

Can you please tone down the notion of wound healing for Figure 6? I appreciate what you are trying to say and I think it is striking that the cells reorient their basal stress fibers into the direction of the mutant cells, form lamellipodia and move into the space of the mutant cell. Nevertheless, the neighboring wild type cells may just spread over the free surface of the ECM substrate until they see a new neighbour. So it may be more of a response to free space rather than a 'wound'. I would thus not call it 'active wound healing' until you can show that that this is a general response to 'wounds' in the follicle epithelium.
