# Supplementary material for: Integrins regulate epithelial cell shape by controlling the architecture and mechanical properties of basal actomyosin networks
Source: PLoS Genet. 2020 Jun 1;16(6):e1008717. doi: 10.1371/journal.pgen.1008717 (PMC7263567; doi:10.1371/journal.pgen.1008717)
Supplement: Attachment — Submitted filename: Review.pdf [file pgen.1008717.s029.pdf]

The revisions present a very much improved version of the manuscript. I would still love to see a more detailed cell biological characterization of these novel basal protrusions in integrin mutants and how they arise, as their characterization is probably the most novel contribution of this manuscript to our understanding of integrin function in the follicle epithelium. However, I do agree with the authors much more on the interpretation of the data then it was the case in the first version of the manuscript.

I still have two major request, although they mainly pertain to language and semantic definition:

1. The authors still use the term 'cortical actin' a lot in the paper, even though I assume they actually want to describe these novel basal protrusions. Could you be clear about what you mean and say 'actin protrusions at basal cell-cell junctions' or 'actin at basal cell edges` or compromise on 'basal/cortical actin rich protrusions'. Please always use this term to avoid confusion with the original definition of 'cortical actin'.

   I understand it is semantics, but because cortical is such a loaded term already I think the authors need to be precise in their semantic definition of these new filipodia/actin rich structures. Please remove all 'cortical actin' references for these basal structures and find a more precise term. A few examples where changes are needed are given here:

   - ....We also observed that all mutant cells within the clone, regardless of whether they were surrounded by either control or mutant cells, displayed increased levels of cortical F-actin, suggesting that this phenotype is cell autonomous (Fig.S5A, A'). In addition, accumulation of cortical F-actin was specific to the basal side, as no difference was found apically (n=22, ec=5, Fig.S5B, B'). These results lead us to suggest that, in addition to its role in stress fiber formation, integrins can regulate F-actin redistribution in FCs.
   - ....Consistently, we found that RNAi-depletion of abi in all FCs, using the traffic jam-Gal4 (tj-Gal4) driver (tj>abiRNAi; (Li et al., 2003), abolished the formation of all actin protrusions, including the cortical actin-rich structures formed in mys FCs (n=34, ec=5, Fig.5A-C).
   - ... We found that mosaic clone FCs expressing zip^{DN}-GFP displayed decreased F-actin levels both in the cortex and in stress fibers (n=26, ec=6, Sup. Fig.6A, A'). Furthermore, expression of zip^{DN}-GFP in integrin mutant FCs (mys; tj> zip^{DN}-GFP) restored cortical F-actin levels and basal surface area, even though the number of stress fibers was further reduced (n=26, ec=6, Sup. Fig.6B, B').
   - .... Furthermore, expression of zip^{DN}-GFP in integrin mutant FCs (mys; tj> zip^{DN}-GFP) restored cortical F-actin levels and basal surface area, even though the number of stress fibers was further reduced (n=26, ec=6, Sup. Fig.6B, B').

2. Several times the authors describe that these novel protrusions ‚contact' the medial basal actomyosin fibres. This description creates extremely confusing mental images of the basal cell surface architecture as the (presumably) extracellular protrusions can only

contact the medial plasma membrane overlying the medial actomyosin fibres and not the actomyosin fibres directly. Or am I missing a huge interpretation?
Anyways, this needs to be clarified either by language (i.e. protrusions interact with the medial plasma membrane thus may be regulated or affected by the medial actomyosin fibres) or by a cartoon illustrating how the authors view the basal surface.

Here are examples of the text in which this description appeared:

- a reorganization of F-actin into a new type of membrane protrusions that emanate from the basal cortex, extend into the cell and contact the medial basal actomyosin fibers.
- The new cortical actin-rich protrusions observed in integrin mutant FCs extended towards the cell center and seemed to contact the medial basal actomyosin fibers (Movie S6).
- Furthermore, it also suggests that the symmetrical and periodic basal surface contractions found in mutant cells could occur by means of medial basal actomyosin networks pulling membrane protrusions inwards.
- Thus, these results strongly suggest that the defects in basal surface growth observed in mys FCs are not due to defective stress fibers number but to re-organization of F-actin into cortical protrusions. These cortical actin-rich protrusions may interact with the spare actomysoin fibers found in the middle of the basal surfaces of mys FCs thus leading to increased membrane tension and cell surface reduction upon actomyosin fiber contraction.

Minor points

- ....Furthermore, these new F-actin protrusions are dynamic and changes in total protrusion area correlate with both myosin accumulation in stress fibers and constriction pulses of the cell membrane.

  What do you mean by protrusion area? The definition of this term is not clear from the introduction yet but only become clear in the results section.

- ... we performed live imaging of mosaic S10B egg chambers expressing Resille-GFP and found that the basal surface of control FCs contacting mutant ones actively spread anisotropycally over the mutant cells

  Please describe what you mean by ‘anisotropically’ (spelling!) and what you base you conclusion of ‘active spreading’ on. I find this a strong interpretation.

- ....Finally, cell culture experiments have shown that loss of contact with the ECM mediated by integrins results in programmed cell death. However, we found that elimination of integrins in main body FCs did not induce cell death, as tested using an antibody to cleaved Dcp-1 (ec=14, Sup. Fig.8).

Can you move this to an earlier position in the manuscript? It kills the arc of the paper ending and, actually, it is a control experiment that is already important for many earlier experiments.
