# Supplementary material for: Integrins regulate epithelial cell shape by controlling the architecture and mechanical properties of basal actomyosin networks
Source: PLoS Genet. 2020 Jun 1;16(6):e1008717. doi: 10.1371/journal.pgen.1008717 (PMC7263567; doi:10.1371/journal.pgen.1008717)
Supplement: Attachment — Submitted filename: Response to reviewers.docx [file pgen.1008717.s028.docx]

19th December 2019

**The Editor**

**PLoS Genetics**

MS ID#: PGENETICS-D-19-01440

MS TITLE: “**Integrins regulate epithelial cell shape by controlling the architecture and mechanical properties** **of basal actomyosin networks**

**Reply to reviewers’ comments**

**Reviewer #1**:

The work of Santa-Cruz Mateos et al explores the role of integrins in Drosophila follicle cells. These cells are a great example of stress fibers that can be studied in vivo, with all the power of genetics and imaging brought by this model. Authors used mosaic tissues for a mutation abolishing integrin complex and analyzed its impact on the actomyosin both on fixed and live samples. Although the authors made some nice observations, as it stands, I do not consider that this manuscript reaches the criteria for publication in PLos Genetics. I have several concerns regarding the data or their interpretation.

1) The article suffers of a lack of novelty. Most of the observations confirm existing data, including on the same tissue, both on F-actin cytoskeleton aspect and actomyosin oscillatory behavior. The best illustration of this is given by the title itself, extremely long but with no clear message: that integrins are important in epithelial cells and have something to do with actomyosin does not seem really new. Title also mentions epithelium morphogenesis and homeostasis, but I cannot see where these points are really addressed in this work. Actually, authors have never looked at a tissue scale.

We agree with the reviewer in that the role of cell-ECM interactions mediated by integrins on the organization and dynamics of actomyosin fibers in *Drosophila* follicle cells (FCs) has been explored before (Delon and Brown, 2009; He et al. 2010; Quin et al. 2017). However, although the more accepted view nowadays is that integrins regulate positively the levels and dynamics of actin and myosin in stress fibers in FCs (He et al. 2010; Quin et al. 2017), a previous report supported a role for integrins in this context as negative regulators (Delon and Brown, 2009). Our results reveal that, in fact, integrin elimination leads to a reduction in F-actin levels in stress fibers in FCs, but to an increased in cortical structures. Thus, we propose that integrins not only regulate F-actin levels, as suggested before (He et al. 2010; Quin et al. 2017), but also F-actin distribution, in contrast to what Quin et al implied. Another novel finding of our study is that it shows that the reorganization of F-actin from stress fibers into other types of actin networks found in integrin mutant FCs has an impact on cell shape, as integrin mutant cells display reduced basal surfaces. Finally, we also show that elimination of integrin from FCs has non-cell autonomous consequences for neighbouring wild type cells, leading to the reorganization of their actin cytoskeleton. In summary, we believe our manuscript does reveal new roles for integrins in both actomyosin dynamics and cell shape changes (see below).

Following the reviewer’s suggestion and with the aim of emphasizing the novel findings of the MS, we propose a more concise title.

2) I am also a bit puzzled by some interpretations :
The main novelty would be that integrin modulates other actin populations as cortical actin and “whips” (figs 1 and 3) . Without integrin, cells produce much less stress fibers (fig 2) and more G-actin is available for cortical F-actin and whips, probably explaining why more of these populations is observed. This raise in cortical actin increases cell tension and induces basal constriction. All data point to this interpretation and authors also reach this conclusion in their discussion and their abstract. Thus, it seems a secondary effect of integrin loss and it is therefore difficult to claim, as authors did several times, that integrin “modulates” or “controls” cortical actin.

The reviewer is right. Since we agree with their interpretation, we have eliminated from the text claims pointing to a role of integrins as regulators of cortical actin.

It would be actually more logical to present the effect of integrin on stress fibers (fig 2) in first. Moreover, whip-like structures are so far poorly characterized at the functional level and depends on the same actin regulators than cortical F-actin; these data could be fused with the ones of fig3. Overall, the figures could be simplified as they show many redundancies. For instance, there are five pictures of F-actin in mys clones around stage 8-9 on four different figures. One could advice the authors to change their manuscript in a shorter report with four figures, highlighting what is really new.

As suggested by the reviewer, we have simplified the figures to show the effect of integrins on stress fibers on Fig.1.

Also, authors stated that wild-type cells next to the mutant ones “acquire spreading capacity” and compared this property with wound healing. It seems highly speculative and even maybe contradictory with their results. Authors have shown that cortical tension increases in mutant cells and that it is associated with basal surface reduction. It is likely that this basal constriction pull on the adjacent cells because of cell-cell adhesion and that stress fibers point towards the mutant cells as a consequence of this mechanical stress. If the wild-type cells were actively spreading over the mutant ones, it would somehow press them, which should decrease cortical tension in the mutant cells. Thus, it seems clearly different than a wound healing process. These two points are really important because the main integrin primary function described so far in these cells is to allow basal surface contraction, at least transitorily. Thus, if not explained properly it might introduce confusion in the field.

We thank the reviewer for this suggestion. Indeed, mechanical stress is an alternative explanation for our results. Thus, in the revised version of the manuscript, we have proposed, both in the Results and Discussion sections, that the reduction in the basal surface observed in late S10B mutant FCs could arise from the activation of spreading capacity and/or a mechanical response in surrounding wild type cells. We also agree with the reviewer in that comparison of this phenomenon with wound healing is highly speculative, something that has also been raised by the other referees. Thus, we have eliminated it.

3) An other important drawback is about the quantitative data throughout the paper.
- Some are missing. Fig 1: whip-like structures are not quantified.

We have provided quantification for all data shown in the paper, including whip-like structures.

Fig 6 : I really like these observations but there is no indication of cell frequency losing their basal domain

This has been quantified. As the basal side of mutant cells is in many cases difficult to visualize, we have first focused on the apical side of the FE and identified mutant cells by the absence of GFP. We have then moved to the basal plane and quantified the number of mutant cells in which the basal surface was hardly visible. This occurs in 70% of mutant clones analysed.

and how frequent the reorientation of the surrounding is.

We have quantified this and showed it occurs in 80% of the cases analysed.

It is also true for several supplemental figures ( S1,S2 and S8 for instance).

- For some of them, no statistic test is provided : 5D, 5E, 4G.

This was a mistake as we had all quantitative and statistical data, which we have now incorporated.

- For some their description is incomplete or approximative and does not allow a clear interpretation. Fig 2D and E: How authors can discriminate long protusions of cortical actin versus stress fibers, especially in mutant cells where less (no?) stress fibers are present and in which protusions are more preeminent due to the increase of cortical actin (visible on fig 1D for instance).

The cortical protrusions can be spatially separated from stress fibers, as the former ones emanate from the cortex, while the latter ones localize to medial positions. To quantify stress fibers, we have chosen S9 FCs cells since, at this stage, mutant FCs still display visible, quantifiable stress fibers and the basal surface is not completely reduced. We have then outlined the basal surface of the cell, excluding the cortical region, and measured fluorescent intensity along a line extended across its central region and traversing the actin bundles (see Material and Methods). We have also performed new in vivo analyses to better characterize the organization and behaviour of the dense cortical F-actin found in integrin mutant FCs (new Fig.2). Time-lapse imaging has resolved this increased cortical F-actin into a new type of cortical actin-rich membrane protrusions that: 1) emerge uniformly from the cell membrane, 2) project towards the center of the cell and 3) do not contain myosin (Movie S5 and 6, new Fig.2). They differ from both, whip-like structures -which show flagella dynamics but not protrusive activity- and from the filopodia associated to egg chamber rotation -which are restricted to the leading edge and reach out over the basal surface of an adjacent cell-. Importantly for the conclusions of our work, these new cortical actin-rich protrusions observed in integrin mutant FCs seem to extend towards the cell center and seem to contact the medial basal actomyosin fibers (Movie S6). In addition, simultaneous quantification of myosin level oscillations, total area occupied by actin-rich protrusions and total basal surface area over time shows that myosin accumulation correlates with both increased protrusion area and reduced basal surface area in mutant FCs (new Fig.2). These results have led us to suggest that the symmetrical and periodic basal surface contractions found in mutant cells could occur by means of medial basal actomyosin networks pulling membrane protrusions inwards. Finally, we find that blocking the formation of protrusions in integrin mutant FCs is sufficient to rescue both the increase in membrane tension and the reduction in basal surface. Altogether, our results strongly suggest that the defects in basal surface expansion observed in integrin mutant FCs most likely result from the increase in membrane tension, produced by the ectopic actin protrusions, rather than a consequence of defects in stress fibers. All these new data have been incorporated in to the text.

Fig 3 : According to the graphs, laser cut data look very robust. However, no pictures or movies are shown. Moreover, the genotype used for these experiments is not indicated.

We have added movies (Movies S7 and S8) and pictures (New Fig.3) from laser ablation experiments carried out in both, control egg chambers expressing Resille-GFP and mosaic egg chambers containing *mys* FC clones and expressing Resille-GFP.

Also, it is not described how the choice of the cut is made in a mutant clone.

I guess the bound is between two mutant cells but what about the two neighboring cells at bound extremities: are they wildtype? are they mutant ? are the results the same in both cases ?

To minimize potential effects due to an anisotropic distribution of forces in the FE, cuts were all made perpendicular to the AP axis in control and mutant FCs located in the central region of fifteen independently cultured egg chambers. Ablations were performed at cell bonds between two control cells (control-control) and two mutant cells (mutant-mutant). Following the reviewer´s suggestion, we have also ablated bonds between a mutant and a control cell (mutant-control) (new Fig.3). Our results show that tension at the boundary between two mutant adjacent cells is higher than that found at boundaries between two adjacent control cells or a control and a mutant cell. We propose this could be a consequence of actin protrusions from two mutant adjacent cells pulling in opposite directions.

- For some quantitative data, the results seem not consistent with other author observations : How authors can measure basal surface with such a small error bar at stage 10 (fig 4G) whereas this value is highly variable during this stage and can even reach zero for some cells according to fig 6.
This was due to the fact that Fig.4 showed the average and standard error of basal surface area measurements in a big sample. We have now used a box plot to show data distribution, which we believe represents better the variability of basal surface size, in agreement with what is shown in Fig.6.

4) Finally, some experiments could be added to fulfill their study. Authors nicely show that Abi RNAi rescues cortical actin levels and basal surface. Laser cuts on such phenotype could fulfill the demonstration that cell surface diminution is due to the increase of cortical actin tension.

We have performed this experiment and found that indeed expression of *abiRNAi* in *mys* FCs rescues membrane tension (Fig.5E).

Moreover, Do they observe wild-type adjacent cells reorienting their stress fibers around mys, Abi RNAi cells. If not that will tend to confirm that it is a response to the mechanical stress induced by mys mutant cells.

We have looked at this and we do not see reorientation of stress fibers in control cells surrounding *mys, abi* RNAi cells. Unfortunately, we believe this result is not conclusive due to the strong disorganization of basal actin bundles caused by *abi* RNAi expression. We have added this comment to the revised version of the manuscript.

Minor points :
Delon and Brown nicely described some changes in integrin complex composition depending on the stage. Could these differences explain why the mutant cells lose their contact specifically at stage 10?

αPS2 mRNA is first detectable at S10B, while αPS1 mRNA is detected from S4-S11. As we observed phenotypes from S7, we believe most of the functions here described are mainly performed by PS1 integrins.

Introduction : “In summary, F-actin organizes in three different types of networks at the basal side of FCs. “ This sentence is difficult to understand as the paragraph mainly describes stress fibers and that the two others populations are mentioned briefly and much earlier. Actually, a scheme explaining the different actin populations could help.

We have changed the sentence and have added a scheme illustrating the different actin populations (Fig.1A).

The only argument of the authors may have for a direct impact of integrin on cortical actin is on fig1C where a localization at, or close to, tricellular junctions is observed. First, this localization of integrin at tricellular junction has already been shown in Schotman et al 2008, in which authors demonstrated that this localization is more a step of their trafficking than a functional site. Then, if such localization will have something to do with the cortical actin defect observed in mys clones, then it should be observed before stage 10. Is it the case?

It is true that integrin localization has already been shown by Schotman et al. Thus, we have removed the picture showing this data and cited Schotman et al 2008.

Some pictures do not seem oriented according to AP axis, as it is usually the case (fig 2 for instance). This is a bit disturbing as stress fibers are normally planar polarized in this tissue.

Amended.

Authors stated that MyoII signal is not increased in mutant cells fig S1 and movie S2. Despite the fact that there is no quantification, I do even see less, probably due to the absence of stress fibers where MyoII localized during the rotation process. To this respect Viktorinova et al, 2017 should be cited.

Yes, myosin levels are reduced and we have added a quantification of this phenotype.

Fig 2C: F-actin looks extremely weird in WT cells. The fact that they have been generated by STED cannot explain such a difference with usual confocal images.
We have changed panel 2C for a normal confocal microscopy image, such as the ones shown in panels 2C and 2D.

Authors tried to detect apoptosis with cleaved cas3 staining. It is now well established that dcp1 staining is a more reliable marker in fly.

This was a mistake in our previous submission, as the original marker used was an anti-dcp1 antibody. We apologize for overlooking this. We have changed this in the text and in the figure.

In the pictures there is a mix of different stages that make very difficult for a non-specialist; Why authors look at stage 8, 9 or 10 (but not the others)?

We have analysed each particular actin network at the stage in which it is better visualized. Stages analysed are now clearly specified in all figures.

Lack of figure annotation makes sometimes article difficult to read :
Fig 2E and 3E: the two graphs represent in appearance the same thing with opposite results. - Fig 3C, 4C : what is plotted on the x-axis?

Amended.

- S3G : what is plotted on the x-axis? What is the difference between the two graphs ? is it time ? if so I do not understand why MyoII and actin would be anticorrelated (whereas they were correlated in the precedent simulations published by the same group.)

The difference between the two graphs is that one represents the real data of the changes in F-actin intensity in control (D) and *mys* (E) FCs and the other one represents predictions from our model (D, E, Valencia Expósito et al. 2016). However, following reviewers´ and editor´s advice to trim down our manuscript, as much as possible, to novel results, we have reduced the section related to the role of integrins on actomyosin oscillations. Thus, we have removed the data where we challenge our model (S3F-H and S4F-H).

Beginning of page 12 : “(or Fat)”?

We are sorry but we are not sure what the reviewer refers to in this comment.

Discussion : Qin paper that showed by optogenetics that integrin are not required for the oscillatory behavior of the cells should be mentioned in the discussion.

Amended.

Actually, I would recommend the authors to show their data on MyoII pulsation in the main figures. Although it comes as confirmation, I find amazing to see that, even in **null** mutant cells, such oscillation still occurs.

We agree with the reviewer in that this is an interesting data. However, as mentioned above, we have condensed all data regarding actomyosin regulation by integrins and transferred most of it to supplementary files.

**Reviewer #2:**

Summary:
This study characterizes the effects of the mysXG43 mutation on the FC cytoskeletal elements – cortical actomyosin fibers, stress fibers, and the whip-like structures at the TCJs, expanding on the knowledge of integrins and their functions in the FE. Previously shown to have increased F-action levels in general, the integrin deficient mutant mysXG43 is now shown to increase F-actin specifically at the whip-like protrusions at the TCJs and the cortical actomyosin network, but NOT at the stress fibers. Integrins are reported to be involved in the setting up, functioning, and maintenance of the cortical actomyosin network as the egg chambers develop from stage 8 – 10. By laser ablation of cell-cell contacts in mutant mosaic FE, they show that the cell membranes are at a higher tension in the mutant cells than in the WT cells. Finally, they show that integrins work with Abi to expand the basal surface area of the endocycling FCs. In the mosaics, they also report a rearrangement of the mutant cells with reduced (or almost absent/sunken) basal membrane, between the WT cells in the FE. This study increases our understanding of the role of integrins in maintaining and regulating the FE actomyosin dynamics.

General comments-
- The result section has a lot of ‘introduction’ at the beginning of each sub-topic, and can be moved to the intro section of the paper to make the data easier to follow.

We have reduced the introductions at the beginning of result sections, going more straight to the results.

- Discussion section has a lot of grammatical errors. Proofread to improve. Good section, though.

We are sorry about this. We hope this has now been corrected.

- The title mentions ‘epithelia morphogenesis and homoeostasis’ but I don’t really see any evidence for this in the data. There is no real evidence for this claim – yes, the mutant cells have an altered morphology (smaller basal surface) but it is the WT cells that compensate for this, and integrins are not shown to have a role in it. And yes, integrins regulate the cytoskeleton and therefore cell shape, but not epithelial morphogenesis per se. Consider rewording so as to not appear to be misleading.

We have now changed the title according to reviewer´s suggestion (see also our reply to comment 1 of Reviewer 1).

- Figure legends also need proofreading to make them more clear – especially the supplementals.

Amended.

- Egg chamber stages for ALL figures need to be specified – even in the supplementary figures.

Amended.

-The movies are awesome.

Specific comments –
- Page 4 – Introduction, paragraph 3 – ovarioles are described as ‘tube-producing eggs’. Please rephrase.

Amended.

- Figure 1 A-A” – What is the purpose of showing the localization of integrins at stage 10a? Make a note of why this is important either here or in the discussion.

We have now removed this information, as it has already been published.

- Is the F-actin stained by phalloidin in stage 8 egg chambers in Fig 1D-D” basal cortical actin, and stress fibers in Fig 2 A-A’? There is a need to distinguish between the two and how you tell them apart, at least in the introduction. Otherwise it gives the impression of conflicting data between your two figures.

We agree with the referee in that data shown in figures 1 and 2 was a bit confusing. We have changed this panel to better show the different actin organizations. We have also added a scheme explaining the different actin populations. In addition, we have added a detailed characterization of the increased basal cortical actin found in integrin mutant cells. Please, see our response to comment 3 to reviewer 1 concerning actin protrusions.

- Page 9 – under “Integrins regulate cortical F actin levels and tension” – the first sentence either needs a reference or can be removed altogether. You can go straight to describing your experiment/data.

We have added a reference.

- Page 10 – under “Morphological consequences…” – The first paragraph contains an excessive amount of introduction or background that is hard to read and, again, distracts from the actual data. Condense for better flow, and for a clearer storyline.

We have shortened the first paragraph considerably.

- Page 11-12 – under “Integrins control basal surface…” zipperDN is reported to have reduced stress fiber numbers. Please include a note on what this signifies in this section, and how it fits in your hypothesis, as you did with the Abi RNAi data.

Amended.

- Page 13 – Discussion – paragraph 1 line 6-7 – “…this integrin function is also critical for the changes in cell shape and area underlying epithelia morphogenesis.” Epithelial morphogenesis indicates a different process in drosophila egg chambers at different stages. Which of those is affected in the mysXG43 mutant cells? Or are you referring generally to cell shape changes caused by the mutation to be ‘epithelial morphogenesis’? Please clarify, as this comes off as misleading to some extent.

We refer to egg chamber elongation. This has now being clarified in the text.

- Page 27 – figure legend –figure 5 – “integrins regulate cell surface growth…” Your data indicates that integrins are required for the normal increase in basal surface area – your figure legend is misleading.

Amended.

- Page 27 – figure legend – figure 6 – “Elimination of integrins in FCs induces spreading of the basal surface of control neighboring FCs” This description indicates that it is the mutation that is somehow ‘inducing’ the spreading of the WT cells in the mosaic. I would argue that it is the phenotype created by the mutation that is eliciting the response from the WT cells in the mosaic (potentially to maintain tissue integrity). Please correct this description.

We have changed the title following the reviewer´s advice.

- Page 28 – Figure legend – Movie S2 – “The whip-like structures found in mysXG43 FCs do not contain myosin” is the title, and you go on to say two lines down that it is the same for both control and mysXG43 mutant cells. It is rather misleading to word the title this way – it makes more sense to say that the mutation does not affect myosin distribution in the whip-like structures. Please correct.

We have changed the title to “Dynamics of whip-like structures in control and *mys* FCs”

- Page 29/Movie S5 – The movie is great! However, it would be easier to see the basal domain of the WT cells expanding if there was an arrow or arrowhead to point out where to look. (The control in Movie S6 helps).

An arrow has been added.

Also, there is a cell doing a lot of moving, almost like a junction remodeling event on the top left corner – please address that with a hypothesis or speculation.

We have occasionally seen this type of cells in our movies. We believe they could be macrophages. In any case, we think there is no relation between this type of cells and the phenotype we described, as they do not appear in other movies we have analysed, such as Movie S4. Thus, to avoid misperceptions, we have amplified the region of the egg chamber in a way that excludes “the cell doing a lot of moving”.

- Page 30 - figure legend – supplementary figure 5 – “…specific of the basal surface” do you mean restricted to the basal fibers?

Yes.

**Reviewer 3**

This manuscript investigates the role of integrins in the organization of basal actomyosin structures, specifically that of basal stress fibers, tri-junctional whips, cortical cytoskeleton. The authors conclude that loss of myospheroid (βPS subunit) successively eliminates basal stress fibres between stages 8 and 10 in Drosophila egg chambers. This loss redistributes actin into tri-junctional whips and the cortical cytoskeleton. The stronger accumulation of actin in the cortical cytoskeleton is suggested to promote enhance cortical contractility driving basal surface reduction. The shrinkage of the basal surface correlates with reorientation of neighboring wild type cells towards *mys* mutant cells and occupancy of the free basal space.

The paper proposes a really interesting idea (shift in basal actin dynamics upon mys loss of function) and follows this idea using state-of-the-art tools (imaging, quantification, genetics). The authors test this idea in a complex developmental context in vivo and have generate beautiful live imaging movies to back up their conclusions.

General comments

While I really appreciate the concept of this idea and I think that the data presented is of high quality, I have one large concern that I think needs to be addressed: I am not convinced that what the authors describe to be ‘cortical actin’ is indeed ‘cortical actin’. The interpretation of these ‘cortical’ structures, however, underlies large conclusions in this manuscript and thus needs to be more clearly validated.

All images of mys mutant clones, the author describe actin structures at basal cell-cell junctions, which they claim are ‘contractile cortical actin’. They base this conclusion on (1) very basal confocal sections and (2) changes in recoil velocity of basal cell-cell junctions.

I am not sure that these structures are cortical and that they can explain the change in basal contractility observed. To me **these actin structures look more like basal filipodia extensions**, rather than a contractile actin structure on bicellular junction. So, I have a hard time reconciling these structures with a change in basal contractility at a mechanistic level. Also, I think they do not appear to contain (more) Sqh/Myosin to explain higher levels of contractility. Also, even though the levels of these ‘cortical’ structures correlate with basal contractility in the functional experiments, I am not convinced that they are causative of the cortical contractile changes (rather than coregulated).

In short: I would love to see at least a better description of the ‘cortical’ structures (what does the basolateral cell cortex look like? Are these true intracellular actin structures or filipodia extension? Is there Myosin on those?) at the basal cell-cell junction and, ideally, better functional evidence that they cause bicellular junction contractility at the basal side.

We have performed new in vivo analyses to better characterize the organization and behaviour of the dense cortical F-actin found in integrin mutant FCs (new Fig.2). In agreement with this reviewer´s comment, time-lapse imaging has resolved this increased cortical F-actin into a new type of cortical actin-rich membrane protrusions. A complete description of these new cortical actin protrusions and their contribution to tension and cell shape defects found in integrin mutant cells is described in the revised MS (see also our reply to comment 3 of Reviewer 1 concerning to actin protrusions).

Minor comments:

‘...Here, we found that integrins also accumulated cortically, localizing with proteins associated to cortical F-actin, such as spectrins (Fig.1C, (Ng et al., 2016). Finally, integrins were also found highly enriched at tricellular junctions (arrowhead in fig.1C)....’ Not sure what the significance of this observation is this early on in the paper. To me the localization in tricellular junctions was never really linked to any of the observations in the paper. I think it should somehow link to the tricellular whips, but that has not been spelled out.

We have removed this data as it has already been described in Schotman et al 2008.

‘...However, the increase in F-actin observed in mysXG43 FCs seemed associated with the whip-like structures found at tricellular junctions rather than with the stress fibers...’ To me the actin structures in Fig 1D look like basal actin stress fibers and not tricellular whips! Can you please show a different image that matches your description of lost stress fibres? I think this is important for Figure 1D.

We have now changed the image.

‘...propel contrary to the direction of egg chamber rotation (Movie 1)...’ • should this be ‘propeling’ ?

Amended.

‘...we challenged our previous model and tested if reduced F-actin levels could lead to shorten and more stochastic oscillations, as well as reduced myosin levels (Valencia- Exposito et al., 2016). We found this was the case (Fig.S3F-H), proving the robustness of the model...’ I do not get this sentence. What model did you test and how does this prove that reduced actin levels alone (rather than lack of mys) are sufficient to shorten oscillations?

We challenged our model on actomyosin oscillations published in Valencia- Exposito et al., 2016. However, following reviewers´ and editor´s advice to trim down our manuscript, as much as possible, to novel results, we have reduced the section related to the role of integrins on actomyosin oscillations, as this has been previously analysed. In this context, we have removed the data where we challenge our model (S3F-H and S4F-H).

‘...Adherent mouse embryonic fibroblasts that gradually detach from the substrate redistribute their F-actin from stress fibers to a more cortical position...’ Reference for this statement is missing... ‘...

Amended.

We found elevated levels of cortical F-actin in the mutant FCs compared to controls

(Fig.3A, A’)...’ See my comment at the beginning but: How do you know that this is cortical and not just a bunch of whips? Many of the bicellular junctions do not show cortical enrichment. The section is very basal. How do you define cortical actin?

See response above.

‘...To do this, we generated large clones and compared cortical F-actin in mutant cells surrounded by either control or mutant cells. We found that cortical F-actin levels were increased in all mutant cells, suggesting that the defects in cortical F-actin due to elimination of integrins are cell autonomous (Fig.S5B, B’)...’ Why do you not show this large clone in the main figure? It immediately answers the question of autonomy (even though I still think that these a filipodia or whip structures and not ‘cortical’ F-actin).

- We have followed this suggestion and now the large clone is shown in the figure.

Figure 3C , What is the x-axis of that graph? Label is missing.  ‘...

- Labels to all axes have now being revised and amended.
- These results demonstrate that cell membranes are under higher tension in integrin mutant cells. Altogether, these results strongly suggest that integrins regulate basal cortical F-actin levels and cortical tension in FCs...’  Laser ablation shows higher tension on these junctions but there is no extra myosin there. Is there more myosin on the cortical junctions to explain higher recoil? Also, the recoil is along the vector of the membrane but the filaments appear orientated perpendicular to it. How can they pull to generate higher tension?

Analysing the dynamics of new type of actin rich protrusions found in integrin mutant cells, we have found a correlation between myosin accumulation, increased protrusion area and basal surface reduction observed in *mys* FCs. These results have led us to suggest that the symmetrical and periodic basal surface contractions found in mutant cells could occur by means of medial basal actomyosin networks pulling membrane protrusions inwards. A direct outcome of this hypothesis is that membrane tension between two adjacent mutant FCs should be higher than the tension between two wild type cells or between a wild type cell and a mutant one, as protrusions from two mutant adjacent cells would be pulling in opposite directions. New laser ablation experiments shown this to be the case. This new data has been incorporated in the text.

Figure 5C  , What is the x-axis of that graph? Label is missing...

- The graph has been changed.
- Can you please tone down the notion of wound healing for Figure 6? I appreciate what you are trying to say and I think it is striking that the cells reorient their basal stress fibers into the direction of the mutant cells, form lamellipodia and move into the space of the mutant cell. Nevertheless, the neighboring wild type cells may just spread over the free surface of the ECM substrate until they see a new neighbour. So it may be more of a response to free space rather than a ‘wound’. I would thus not call it ‘active wound healing’ until you can show that that this is a general response to ‘wounds’ in the follicle epithelium.

We agree with the reviewer in that comparison of this phenomenon with wound healing is highly speculative, something that has also been raised by the other referees. Thus, we have eliminated it. We agree with the reviewer in that the reorientation of the actin cytoskeleton observed in wild type cells surrounding mutant ones could be a response to free space. We have clarified this in the text with the following sentence: “In this context, wild type FCs surrounding mutant ones could sense free ECM space left by the mutant cells and respond by spreading and reorganizing their actin cytoskeleton”. In addition, following Reviewer 1´s suggestion, we have considered a second explanation for this behaviour. In this context, the interactions of the actin rich protrusions formed in mutant FCs with medial basal fibers would pull their membranes inwards generating a mechanical stress in surrounding wild type cells. This could would lead to their stretching and stress fiber reorganization. Both explanations have been added in the revised MS.
