# Supplementary material for: Integrins regulate epithelial cell shape by controlling the architecture and mechanical properties of basal actomyosin networks
Source: PLoS Genet. 2020 Jun 1;16(6):e1008717. doi: 10.1371/journal.pgen.1008717 (PMC7263567; doi:10.1371/journal.pgen.1008717)
Supplement: Attachment — Submitted filename: Response to Reviewers 2nd Revision.docx [file pgen.1008717.s030.docx]

**Response to reviewers**

**Reviewer #1:** The manuscript is improved but I am still not fully convinced about the novelty and the relevance of this work. I guess the question is whether the characterization of secondary effects of integrin loss can be considered as “new roles for integrins” as authors claim in their rebuttal.

1) I still do not find the title in line with the paper as it is presented: the title mentions actomyosin whereas myosin is not looked at anymore in the main figures, which I think is unfortunate.

We disagree with the reviewer in that “the title is not in line with the paper as the title mentions actomyosin while myosin is not looked at anymore in the main figures”. In fact, Figure 2 shows simultaneous quantification of **oscillations of** **myosin levels,** total area occupied by actin-rich protrusions and total basal surface area over time, revealing that **myosin** accumulation correlates with both increased protrusion area and reduced basal surface area in mutant FCs. These results have led us to suggest that the symmetrical and periodic basal surface contractions found in mutant cells could occur by means of medial basal **actomyosin** networks interacting with membrane protrusions, which we propose could lie behind the defects in basal surface expansion observed in integrin mutant cells. In addition, there are three Movies (Movies S2, S4 and S6) and three Supplementary figures (Sup. Fig.1, 2 and 4) demonstrating that integrins regulate **myosin** organization and dynamics. Thus, we believe mentioning **myosin** in the title is adequately justified.

2) Authors wrote:

“In addition, we noticed that the basal stress fibers, which are randomly oriented in wild type S10B FCs (yellow asterisks in Fig.6E, E’, Delon and Brown, 2009), polarized towards the mutant cells in control FCs surrounding mys FCs (n=15, ec=8, yellow arrows in Fig.6E, E’). Quantification of this phenotype revealed that control cells re-orient their stress fibers around clones of mutant cells in 80% of the cases analysed (n=15, ec=9). “

As I understand it, the same quantification is given twice but not consistently …

Moreover few lines before authors indicate that “ Our analysis of later egg chambers (stage 10b) showed that 70% of the mutant clones (n=30, ec=8) contained cells in which the basal area was hardly visible (Fig.6A-B’) “

But then why number are different (30 in one case 15 in the other) and to what the 80% refer: is 80% of the 70% or 80% of the totality? It may change quite a lot the interpretation.

We agree with the reviewer in that the data is a bit confusing as it is described. These are two different, although related, phenotypes. On one hand, we have analysed and quantified basal area expansion in mutant FCs compared to controls. To do this, we only scored mutant FCs presenting an extremely reduced basal surface, and we found this to occur in 70% of mutant clones analysed. On the other hand, we analysed re-orientation of basal stress fibers in wild type FCs surrounding mutant clones and found this to happen in 80% of cases analysed. Furthermore, as re-orientation of stress fibers was always observed in wild type cells surrounding mutant ones with extremely reduced basal surface, we believe the two phenotypes might be related. In any case, we have now changed the text to clarify this point.

3) laser cuts movies are nice but on the provided examples I cannot see the difference in the recoil between the two genotypes. Also the duration of the movies and the image size (different scales ?) are different, making theme difficult to compare.

We have adapted the movies so that they now have the same duration and image size.

4) Fig 5E : values of recall velocities for WT/WT cells and mys/mys cells look identical to the ones of Fig 3C. If the authors reused the same data, it should be indicated. Moreover the n value is not indicated for mys, abi RNAi cuts.

We have not reused the data showing the initial velocity of vertex displacement between two adjacent wild type or *mys* FCs. They look identical because in fact they are very similar, 0.58μm/sec (Fig.3C) and 0.59μm/sec (Fig.5E) for control/control cells and 1.26μm/sec (Fig.3C) and 1.28μm/sec (Fig.5E) for *mys/mys* cells. The n for *mys;abiRNAi* cuts is n=15, ec=15. This data has now been included in the revised version of the manuscript. In addition, the numerical data underlying these graphs is now available in a folder called Numerical data underlying graphs.

5) Whether Abi RNAi could rescue tension increase and stress fiber orientation in neighboring cells should have tested using MARCM approach. 1) it would have clearly shown that the effect on tension is cell autonomous 2)The general effect of Abi RNAi on stress fiber orientation because it blocks rotation could have been anticipated (already published) to choose a better strategy.

We did try the MARCM approach to try to generate *mys* mutant FC clones expressing *abiRNAi*. Unfortunately, the size of *mys* clones we could obtain using this technique was very small, which did not allow us to properly visualize in vivo the spreading of wild type FCs over the mutant ones.

6) Image quality of the PDF is poor, making very difficult to look at some figures.

We agree with the reviewer in that image quality of the PDF is poor, we will try to upload figures at maximal resolution.

**Reviewer #2:** The responses to the original comments are acceptable. However, the manuscript still contains many grammatic errors and needs to be proofread thoroughly.

We are sorry about this. We will go throughout the paper again looking carefully for grammatical errors and will try to correct all of them.

**Reviewer #3:** review is uploaded as attachment.

The revisions present a very much improved version of the manuscript. I would still love to see a more detailed cell biological characterization of these novel basal protrusions in integrin mutants and how they arise, as their characterization is probably the most novel contribution of this manuscript to our understanding of integrin function in the follicle epithelium. However, I do agree with the authors much more on the interpretation of the data then it was the case in the first version of the manuscript.

I still have two major request, although they mainly pertain to language and semantic definition:

1. The authors still use the term ‘cortical actin’ a lot in the paper, even though I assume they actually want to describe these novel basal protrusions. Could you be clear about what you mean and say ‘actin protrusions at basal cell-cell junctions’ or ‘actin at basal cell edges` or compromise on ‘basal/cortical actin rich protrusions’. Please always use this term to avoid confusion with the original definition of ‘cortical actin’.

I understand it is semantics, but because cortical is such a loaded term already I think the authors need to be precise in their semantic definition of these new filipodia/actin rich structures. Please remove all ‘cortical actin’ references for these basal structures and find a more precise term. A few examples where changes are needed are given here:

• ....We also observed that all mutant cells within the clone, regardless of whether they were surrounded by either control or mutant cells, displayed increased levels of cortical F-actin, suggesting that this phenotype is cell autonomous (Fig.S5A, A’). In addition, accumulation of cortical F-actin was specific to the basal side, as no difference was found apically (n=22, ec=5, Fig.S5B, B’). These results lead us to suggest that, in addition to its role in stress fiber formation, integrins can regulate Factin redistribution in FCs.

• ....Consistently, we found that RNAi-depletion of abi in all FCs, using the traffic jam-

Gal4 (tj-Gal4) driver (tj>abiRNAi; (Li et al., 2003), abolished the formation of all actin protrusions, including the cortical actin-rich structures formed in mys FCs (n=34, ec=5, Fig.5A-C).

• ... We found that mosaic clone FCs expressing zipDN-GFP displayed decreased Factin levels both in the cortex and in stress fibers (n=26, ec=6, Sup. Fig.6A, A’). Furthermore, expression of zipDN-GFP in integrin mutant FCs (mys; tj> zipDN-GFP) restored cortical F-actin levels and basal surface area, even though the number of stress fibers was further reduced (n=26, ec=6, Sup. Fig.6B, B’).

We agree with the reviewer in that cortical is a loaded term and it might lead to confusion. Thus, following the reviewer´s suggestion, we have changed all “cortical actin” references for a more precise term, such as “F-actin at basal edges” or “basal actin rich protrusions”.

2. Several times the authors describe that these novel protrusions ‚contact‘ the medial basal actomyosin fibres. This description creates extremely confusing mental images of the basal cell surface architecture as the (presumably) extracellular protrusions can only contact the medial plasma membrane overlying the medial actomyosin fibres and not the actomyosin fibres directly. Or am I missing a huge interpretation? Anyways, this needs to be clarified either by language (i.e. protrusions interact with the medial plasma membrane thus may be regulated or affected by the medial actomyosin fibres) or by a cartoon illustrating how the authors view the basal surface.

Here are examples of the text in which this description appeared:

• a reorganization of F-actin into a new type of membrane protrusions that emanate from the basal cortex, extend into the cell and contact the medial basal actomyosin

fibers.

• The new cortical actin-rich protrusions observed in integrin mutant FCs extended towards the cell center and seemed to contact the medial basal actomyosin fibers (Movie S6).

• Furthermore, it also suggests that the symmetrical and periodic basal surface contractions found in mutant cells could occur by means of medial basal actomyosin networks pulling membrane protrusions inwards.

• Thus, these results strongly suggest that the defects in basal surface growth observed in mys FCs are not due to defective stress fibers number but to reorganization of F-actin into cortical protrusions. These cortical actin-rich protrusions may interact with the spare actomysoin fibers found in the middle of the basal surfaces of mys FCs thus leading to increased membrane tension and cell surface reduction upon actomyosin fiber contraction.

The reviewer is right in that we cannot use the term “contact” to describe the behaviour of the actin-rich protrusions in relation to the medial basal actomyosin fibres. Thus, we have used more appropriated terms and/or expressions, such as “cortical actin-rich protrusions overly medial basal actomyosin fibers” or “cortical actin-rich protrusions could be regulated by the medial basal actomyosin fibers”.

Minor points

• ....Furthermore, these new F-actin protrusions are dynamic and changes in total protrusion area correlate with both myosin accumulation in stress fibers and constriction pulses of the cell membrane.

What do you mean by protrusion area? The definition of this term is not clear from the introduction yet but only become clear in the results section.

We measured protrusion area from in vivo movies as performed in Fernandez-Espartero et al. 2013 and in Sanchez-Sanchez et al. 2017. In brief, protrusion area was calculated in FCs expressing the membrane marker Resille-GFP by drawing freehand Regions of Interest (ROIs) from the cell surface to the tip of the protrusions, as shown in orange in Fig.2D. Total protrusion area was then calculated as a percentage of total surface area. We have now added this paragraph to the section Image processing and data analysis in Materials and Methods.

• ... we performed live imaging of mosaic S10B egg chambers expressing Resille-GFP and found that the basal surface of control FCs contacting mutant ones actively spread anisotropycally over the mutant cells

Please describe what you mean by ‘anisotropically’ (spelling!)

By anisotropic spreading we mean polarized spreading of wild type FCs towards mutant ones.

and what you base you conclusion of ‘active spreading’ on. I find this a strong interpretation.

We base our conclusion of active spreading on the fact that we see wild type FCs moving towards neighbouring mutant ones, something that we never observe in the control situation. However, and taking into account that we have considered another possibility to explain this behaviour, i.e. mutant cells might be generating a mechanical stress in will type ones leading to their stretching, we have softened our statement in the text.

• ....Finally, cell culture experiments have shown that loss of contact with the ECM mediated by integrins results in programmed cell death. However, we found that elimination of integrins in main body FCs did not induce cell death, as tested using an antibody to cleaved Dcp-1 (ec=14, Sup. Fig.8).

Can you move this to an earlier position in the manuscript? It kills the arc of the paper ending and, actually, it is a control experiment that is already important for many earlier experiments.

We agree with the reviewer in that this data is an important control and it should go earlier in the paper. Thus, we have moved it to the beginning of the first section of results. With this rearrangement, Fig.S8 is now Fig.S1.
